# Procalcitonin as a predictive marker in COVID-19: A systematic review and meta-analysis

**DOI:** 10.1371/journal.pone.0272840

**Published:** 2022-09-09

**Authors:** Amit Kumar, Era Karn, Kiran Trivedi, Pramod Kumar, Ganesh Chauhan, Aradhana Kumari, Pragya Pant, Murali Munisamy, Jay Prakash, Prattay Guha Sarkar, Kameshwar Prasad, Anupa Prasad

**Affiliations:** 1 Department of Laboratory Medicine, Rajendra Institute of Medical Sciences, Ranchi, Jharkhand, India; 2 Department of Biotechnology, Patna University, Bihar, India; 3 Department of Obstetrics and Gynecology, Rajendra Institute of Medical Sciences, Ranchi, Jharkhand, India; 4 Department of Biochemistry, Rajendra Institute of Medical Sciences, Ranchi, Jharkhand, India; 5 Department of Genetics and Genomics, Rajendra Institute of Medical Sciences, Ranchi, Jharkhand, India; 6 Department of Nephrology, Rajendra Institute of Medical Sciences, Ranchi, Jharkhand, India; 7 Department of Translational Medicine, All India Institute of Medical Sciences, Bhopal, India; 8 Department of Critical Care, Trauma Centre, Rajendra Institute of Medical Sciences, Ranchi, Jharkhand, India; 9 Department of Cardiology, Rajendra Institute of Medical Sciences, Ranchi, Jharkhand, India; 10 Department of Neurology, Rajendra Institute of Medical Sciences, Ranchi, Jharkhand, India; Keele University, UNITED KINGDOM

## Abstract

**Background:**

Coronavirus disease 2019 has emerged as a global pandemic causing millions of critical cases and deaths. Early identification of at-risk patients is crucial for planning triage and treatment strategies.

**Methods and findings:**

We performed this systematic review and meta-analysis to determine the pooled prognostic significance of procalcitonin in predicting mortality and severity in patients with COVID-19 using a robust methodology and clear clinical implications.

**Design:**

We used Preferred Reporting Items for Systematic Reviews and Meta-Analyses and Cochrane Handbook for Systematic Reviews of Interventions guidelines. We included thirty-two prospective and retrospective cohort studies involving 13,154 patients.

**Results:**

The diagnostic odds ratio of procalcitonin for predicting mortality were estimated to be 11 (95% CI: 7 to 17) with sensitivity, specificity, and summary area under the curveof 0.83 (95% CI: 0.70 to 0.91), 0.69 (95% CI: 0.58 to 0.79), and 0.83 (95% CI: 0.79 to 0.86) respectively. While for identifying severe cases of COVID-19, the odds ratio was 8.0 (95% CI 5.0 to 12.0) with sensitivity, specificity, and summary area under the curve of 0.73 (95% CI 0.67 to 0.78), 0.74 (0.66 to 0.81), and 0.78 (95% CI 0.74 to 0.82) respectively.

**Conclusion:**

Procalcitonin has good discriminatory power for predicting mortality and disease severity in COVID-19 patients. Therefore, procalcitonin measurement may help identify potentially severe cases and thus decrease mortality by offering early aggressive treatment.

## 1. Introduction

Severe acute respiratory syndrome coronavirus 2 (SARS-CoV-2) infection due to the high transmissibility has led to the global pandemic with the coronavirus disease 2019 (COVID-19) crossing a caseload of 213 million and mortality of 4.44 million mark [1]. Most patients experience a mild course of the disease, but a small percentage develop a severe condition that requires intensive care and mechanical ventilation. Patients with severe disease may progress to pulmonary dysfunction, multiple organ dysfunction, and death [2]. Several biochemical markers like C-reactive protein (CRP), interleukin-6 (IL-6), white cell count, lactate dehydrogenase (LDH), ferritin, albumin, and D-dimer predict COVID-19 outcome [3, 4]. However, none of them is a definitive marker. These biomarkers add objective values to subjective clinical symptoms of COVID-19 such as fever, high temperature, cough, myalgia, headache, diarrhea, dyspnea, acute respiratory distress syndrome (ARDS), and acute cardiac injury [5]. Procalcitonin (PCT) is a commonly used blood biomarker for assessing bacterial infection and disease progression. It is also used to guide antibiotic therapeutic administration in patients with severe respiratory infection and sepsis which results in reduced antibiotic exposure and improved clinical outcomes [6]. PCT ≥ 1 ng/mL and <10% decrease from the previous day signal uncontrolled bacterial infection [7]. However, PCT is also often raised due to the inflammatory cascade caused by a cytokine storm in COVID-19 patients [8, 9]. Emerging evidence suggests that mildly elevated PCT can also help identify COVID-19 patients at higher risk for clinical deterioration [10]. Early identification of at-risk patients can assist clinicians in planning triage and treatment strategies. Multiple studies have mentioned the prognostic significance of PCT in patients with COVID-19 [11]. Previous systematic reviews and meta-analyses have also suggested the prognostic role of PCT [10, 12–14]. A recently published systematic review included 52 research articles but did not perform a meta-analysis due to the lack of uniformity of the statistical models included in the studies [12]. Findings of other published meta-analyses are either limited by the inclusion of a smaller number of studies [10, 14]or by a lack of robust methodology for the presentation of prognostic meta-analysis as per recommended guidelines. Therefore, we aimed to determine the pooled prognostic significance of procalcitonin in predicting the mortality and severity of COVID-19 using a robust methodology. Our meta-analysis includes the reports of pooled sensitivity, pooled specificity, the summary area under the curve, publication bias, GRADE evidence, QUIPS tool for assessing the risk of bias, model validity test, and prospective registry of meta-analysis in PROSPERO to obtain clear clinical implications.

## 2. Methods

We registered the protocol for our meta-analysis with PROSPERO (Registration Number CRD42021248103). In addition, we used preferred Reporting Items for Systematic Reviews and Meta-Analyses (PRISMA) [15] and the Cochrane Handbook for Systematic Reviews of Interventions [16] guidelines to carry out this research work.

### 2.1. Study inclusion criteria

We considered prospective and retrospective cohort studies to predict adverse outcomes in COVID-19 patients. The inclusion criteria were:(a) studies reported an association between blood PCT level and COVID-19 severity/mortality, (b) studies reported adequate data for extraction of the true positive, true negative, false positive, and false negative values to determine the prognostic significance of PCT, (c) Full-text articles available in English. The exclusion criteria were: (a) PCT reported as mean value, and it was impossible to extract the sensitivity and specificity, (b) studies that used a cut-off value of PCT ≥ 1 ng/ml [6], (c) studies published as case reports, case series, review articles, abstract publications, articles with no full text available, or pre-print publications, and (d) studies lacking information on the association of PCT with severity or mortality.

### 2.2. PICO criteria

#### Participants

We included studies with COVID-19 patients who needed hospitalization or intensive care unit (ICU) admission and used the same diagnostic criteria of COVID-19 as reported in the individual studies.

#### Intervention

Studies provided the cut-off value of PCT at the time of admission<1 ng/ml and reported adequate data for the calculation of sensitivity and specificity of PCT for predicting mortality or severity.

#### Comparator

Alive patients for the prediction of mortality and non-severe patients for the prediction of severity were the comparators.

#### Outcomes

The outcomes assessed were mortality and the severity of the disease. The severity of the disease was defined as ICU admission or invasive mechanical ventilation, or as per Chinese Clinical Guidance for COVID-19 Pneumonia Diagnosis and Treatment (7th edition), or WHO interim guidance for COVID-19, or American Thoracic Society guidelines for community-acquired pneumonia [Table 1].

**Table 1:** Characteristics of the included studies.

Author	Year	Study period	Place of Study	Study design	Total case (n)	Mean Age (yrs)	Male (%)	PCT cut-off (ng/ml) at admission	Death (%)	Comorbidities (%)	Outcome
Asghar MS et al.	2020	NA	Pakistan	RC	364	52.69 ± 15.8	67.5	0.12	27.7	NA	Mortality
Bahl A et al.	2020	March 1st to March 31st, 2020	USA	RC	1461	62 ± 17.8	52.7	0.5	25.8	DM: 29.4%; HTN: 51%; HD:1.2%; CKD: 5.1%	Mortality
Berenguer J et al.	2020	Upto 17th April 2020	Spain	Multicentric RC	4035	70 ± 17.8 [56.0–80.0]	61.0	0.5	27.8	DM: 21.8%; HTN:51%; HD: 23.3%; CKD: 5%;	Mortality
Cao J et al.	2020	January 3to Feb 1, 2020	China	PC	102	54 ± 29.63 [37.0–67.0]	52	0.1	20.7	DM: 10.8%; HTN: 27%; HD: 4.9%; CKD: 3.9%	Mortality
Chen R et al.	2020	Upto March 22, 2020	China	Multicentric RC	548	56 ± 14.5	52.8	0.5	17.6; 36.3	DM: 11.1%; HTN: 27%; HD: 6.4%; CKD: 2.4%	Mortality and Severity*
Chen T et al.	2020	January 13 to Feb 28, 2020	China	RC	274	62 ± 19.3 [44.0–70.0]	62	0.05	40.7	DM: 17%; HTN: 34%; HD: 8%; CKD: 1.0%	Mortality
Claudia G et al.	2020	February 26 to April 30, 2020.	Switzerland	RC	99	67 ± 14.8, [56.0–76.0]	63	0.11	18.2	DM: 22%; HTN: 57%; HD: 25%; CKD: 28%	Mortality
Duan J et al.	2020	January 1 to February 29, 2020	China	RC	348	44.0 ± 15.0	52	0.04	5.7	DM: 3%; HTN: 7%; HD: 2%; CKD: 0.3%	Severity *
Goyal P et al.	2020	March 3to March 27, 2020	USA	Multicentric RC	393	62.2 ± 18.6 [48.6–73.7]	60.6	0.5	33.1	DM: 25.2%; HTN: 50.1%; HD: 13.7%; CKD: NA	Severity (use of invasive mechanical ventilation)
Guan WJ et al.	2020	December 11, 2019 to January 29, 2020	China	RC	1099	47 ± 17.04 [35.0–58.0]	58.1	0.5	18.5	DM: 7.4%; HTN: 15%; HD: 2.5%; CKD: 0.7%	Severity***
Herold T et al.	2020	February 29 to April 9, 2020	UK	PC	89	61 ± 17.1 [18.0–84.0]	89	0.05	4.08	DM: 8%; HTN: 50%; HD: 8%; CKD: NA	Mortality
Huang C et al.	2020	Dec 31, 2019 to Jan 1, 2020	China	RC	41	49.0 ± 12.6 [41–58]	73	0.1	30.8	DM: 20%; HTN:15%; HD: 15%; CKD: NA	Severity (ICU admission)
Hu R et al.	2020	January 30, 2020 to March 17, 2020	China	RC	95	57.6 ± 14.7	41.0	0.1	34.7	DM: 13%; HTN: 27%; HD: 8%; CKD: NA	Severity*
Jiang M et al.	2021	January 1 to April 10, 2020	China	RC	1717	63 ± 14.07	48.7	0.05	11.7	DM: 9.9%; HTN: 48%; HD: 7.5%; CKD: NA	Mortality
Jin H et al.	2020	February 9 to March 20, 2020	China	RC	129	65 ± 12.6 [54.0–71.0]	51.9	0.085	17.8	DM: 18.6%; HTN-46%; HD-15.5%; CKD-NA	Mortality
Keski H et al.	2021	NA	Turkey	RC	302	57.1 ± 17.6	49	0.11	8.28	DM: 26.5%; HTN: 38%; HD: 11.3%; CKD: 5.3%	Mortality
Lei S et al.	2020	January 1 to February 5, 2020	China	RC	34	55 ± 14.8 [43.0–63.0]	41.2	0.1	44.1	DM: 23.5%; HTN: 38.2%; HD: 20.6%; CKD: 2.9%	Severity (ICU admission)
Li H et al.	2020	January 18to February 26, 2020	China	RC	132	62.05 ± 12.68	56.8	0.05	54.5	NA	Severity*
Li K et al.	2020	January 2020 to February, 2020	China	RC	83	45.5 ± 12.3	53.0	0.05	30.1	DM: 7.8%; HTN: 6%; HD: 1.2%; CKD: NA	Severity*
Liu F et al.	2020	January 18, 2020, to March 12, 2020	China	RC	140	65.5 ± 13.8 [54.3–73.0]	35.0	0.07	23.6	DM: 24.3%; HTN: 45.0%; HD: 25.0%; CKD-NA	Severity*
Montrucchio G et al.	2021	March to June, 2020	Ialy	PC	57	64.0 ± 12.6 [54–71]	87.7	0.5	54.4	DM: 19.3%; HTN: 54%; HD: 19.3%; CKD: NA	Mortality
Pan F et al.	2020	January 27 to March 19 2020	China	RC	124	68 ± 10.37	68.5	0.2	71.7	DM: 20.2%; HTN: 50%; HD: 15.3%; CKD: NA	Mortality
Qin Z et al.	2020	February 04 to March 04, 2020	China	RC	118	63.1 ± 15.7	41.5	0.078	34.7	DM: 11.9%; HTN: 40%; HD: 16.1%; CKD: 3.4%	Mortality
Rubio-Sánchez R et al	2021	March 14to June 5, 2020	Spain	RC	197	72 ± 18.5 [59–84]	49.2	0.19	35.5	DM: 27.4%; HTN: 58.4%; HD: 28.9%; CKD: 13.7%	Severity (ICU admission)
Sayah W	2021	March 22 to June 16, 2020	Algeria	PC	153	61.0 ± 13.95	67	0.138	24.8; 52.3	DM: 39%; HTN: 32%; HD: 7.0%; CKD: NA	Mortality and Severity**
Vanhomwegen C et al.	2021	March 3 to June 2, 2020	Belgium	RC	66	61 ± 16.3	63	0.5	30.3	DM: 32%; HTN: 51%; HD: NA; CKD: 15%	Mortality
Wang D et al.	2020	January 1^st^ to January 28^th^, 2020	China	RC	138	56 ± 19.3 [42.0–68.0]	54.3	0.05	26.1	DM: 10.1%; HTN: 31.2%; HD: 14.5%; CKD: 2.9%	Severity (ICU admission)
Wang F et al.	2020	January 2020 and March 2020	China	PC	65	57.1 ± 13.03	66.7	0.05	53.8	DM: 16.7%; HTN: 51.9%; HD: 5.6%; CKD: 5.6%	Severity*
Xu J et al.	2020	January 17 to March 2, 2020	China	RC	76	59.11 ± 14.5	60.53	0.1	22.4	DM: 19%; HTN: 35%; HD: 9.2%; CKD: 6.6%	Mortality
Ye J et al.	2021	January to April 2020	China	RC	170	44.7 ± 17.8	65.5	0.15	17.1	NA	Severity*
Yu J et al.	2020	January 24to April 26, 2020	China	PC	314	64.65	57.6	0.795	73.6	NA	Severity*
Zhou F et al.	2020	Dec 29, 2019 to Jan 31, 2020	China	RC	191	56 ± 15.6 [46·0–67·0]	62	0.1	31.1	NA	Mortality

Abbreviations: RC- Retrospective cohort; PC- Prospective cohort; NA- Not available; DM-Diabetes mellitus; HTN- Hypertension; HD- Heart disease; CKD- Chronic Kidney Disease.

* As per Chinese Clinical Guidance for COVID-19 Pneumonia Diagnosis and Treatment (7th edition)[51], Severity defined by Respiratory rate > 30 breaths/min or or SpO2 < 93% or PaO2/FiO2 ratio ≤ 300 or patients whose pulmonary imaging showed progression of lesion >50% within 24–48 hours.

** WHO interim guidance for COVID-19[34]. Severity defined by clinical signs of pneumonia and Respiratory rate > 30 breaths/min; or severe respiratory distress; or SpO2 < 90%[52].

*** American Thoracic Society guidelines for community-acquired pneumonia 2019[53]. Severity defined by three or more minor criteria or one major criteria. Minor criteria include Respiratory rate ≥ 30 breaths/min, multilobe infiltrates PaO2/FiO2 ratio ≤ 250, confusion/disorientation, Uremia (blood urea nitrogen level ≥ 20 mg/dl), Leukopenia* (white blood cell count < 4,000 cells/μl), Thrombocytopenia (platelet count < 100,000/μl), Hypothermia (core temperature < 36°C), and Hypotension requiring aggressive fluid resuscitation. Major criteria include Septic shock with need for vasopressors and Respiratory failure requiring mechanical ventilation.

### 2.3. Study design

The systematic review and meta-analysis included retrospective cohorts and prospective cohorts.

### 2.4. Ethical approval

Not Required.

### 2.5. Search strategy

This study followed PRISMA guidelines 2020. An electronic search was carried out using PubMed, Central, Cochrane Library, and Google Scholar electronic search engine. We applied the English language and human participants filter to the search. We conducted our last search up to May 31, 2021. The following free text words and medical subject heading (MeSH) terms were used ("procalcitonin"[MeSH Terms] OR "procalcitonin"[All Fields] OR "procalcitonine"[All Fields]) AND ("covid 19"[All Fields] OR "covid 19"[MeSH Terms] OR "covid 19 vaccines"[All Fields] OR "covid 19 vaccines"[MeSH Terms] OR "covid 19 serotherapy"[All Fields] OR "covid 19 serotherapy"[Supplementary Concept] OR "covid 19 nucleic acid testing"[All Fields] OR "covid 19 nucleic acid testing"[MeSH Terms] OR "covid 19 serological testing"[All Fields] OR "covid 19 serological testing"[MeSH Terms] OR "covid 19 testing"[All Fields] OR "covid 19 testing"[MeSH Terms] OR "sarscov 2"[All Fields] OR "sarscov 2"[MeSH Terms] OR "severe acute respiratory syndrome coronavirus 2"[All Fields] OR "ncov"[All Fields] OR "2019 ncov"[All Fields] OR (("coronavirus"[MeSH Terms] OR "coronavirus"[All Fields] OR "cov"[All Fields]) AND 1stDecember 2019 to 31stMay 2021. We also scanned the reference lists of the identified studies and relevant reviews on the patients for additional possible studies.

### 2.6. Risk of bias assessment

We evaluated the methodological quality using the QUIPS tool [17], modified for our review. In the “study participation” domain, we marked a high risk of bias in case of an inadequate description of the selection of study participants. In the “study attrition” domain, moderate risk of bias resulted from 5–10% loss to follow-up data and high risk in case of >10% loss to follow-up data. In the “prognostic factor measurement” domain, we judged a high risk of bias if there was inadequate reporting on PCT measurement and if missing PCT data was > 20%. In contrast, we considered a moderate risk of bias if missing data of PCT was 10–20%. Finally, we judged a high risk of bias in the confounding factor assessment if the study did not provide bacterial culture data to exclude bacterial infection. For analyzing the overall risk of bias in the included studies, we made a scoring system in which domains were scored 0 for low, 1 for moderate, and 2 for high risk of bias. After calculating the total domain score of every study, we classified the range of score 0 to 2 at low risk, 3 at moderate risk, and more than 3 at high risk of bias.

### 2.7. Data collection and analysis

Two review authors, AP and EK, independently and repeatedly screened and retrieved all potentially eligible studies separately based on the eligibility criteria described above. The screening of the relevant published papers began with the titles and abstracts and then progressed to the full text if deemed worthy. A third author (AK) settled disagreements, if any, involved in this study. Data were extracted from the individual studies and entered in the excel file independently by two independent authors and verified by the third author. We extracted the following details: the number of participants, place of study, study design, study duration, the cut-off value of PCT, the area under the curve, and outcomes measured. In addition, we contacted the authors twice by email for the missing information in the articles to include the study for computing pooled sensitivity and pooled specificity.

### 2.8. Evidence synthesis

Considering the clinical and statistical heterogeneity, random-effects model was used to calculate the pooled sensitivity and specificity with a 95% confidence interval (CI). After removal of the extreme outlier study, sensitivity analysis was done to obtain homogeneous results. We assessed the publication bias using a combination of asymmetry observed in Deeks funnel plot and the P-value [18]. The cut-off of the P-value for considering significant publication bias was <0.1. We used *I*^*2*^ statistics and Cochran’s Q test [12] to assess the heterogeneity among the studies. P-value of < 0.1 was considered significant. I^2^ represents the percentage of variation explained beyond chance or sampling error. *I*^*2*^ of less than 50% is acceptable for the fixed-effect model, while more than 50% reflects moderate to considerable heterogeneity. The Goodness-of-fit test checked the validity of the model. We used the Grading of Recommendations Assessment, Development, and Evaluation (GRADE) approach to assess the quality of evidence for PCT as a prognostic marker [19].Fagan Nomogram analysis was done to check pre-test and post-test probabilities. We conducted a multivariate meta-regression analysis using STATA command midastpfpfntn, reg(percent_death diabetes hypertension heart_diseasewbc_countneurtrophil_countmean_crp cat_age) to investigate the potential sources of heterogeneity in pooled effect size among included studies [20]. Following relevant variables- diabetes mellitus (in percent), hypertension (in percent), total WBC count, neutrophil count, and mean C-reactive protein, categorical CRP [less than or equal to 5mg/L or more than 5mg/L], and PCT level in ng/ml were considered for meta-regression analysis. The variables which turned significant or clinically relevant were considered for subgroup analysis. P-value <0.05 was considered significant for the effect estimate. We used STATA software version 13.0 (StataCorp. College Station, Texas, USA) to analyze the data.

## 3. Results

### 3.1 Study characteristics

**Fig 1:**
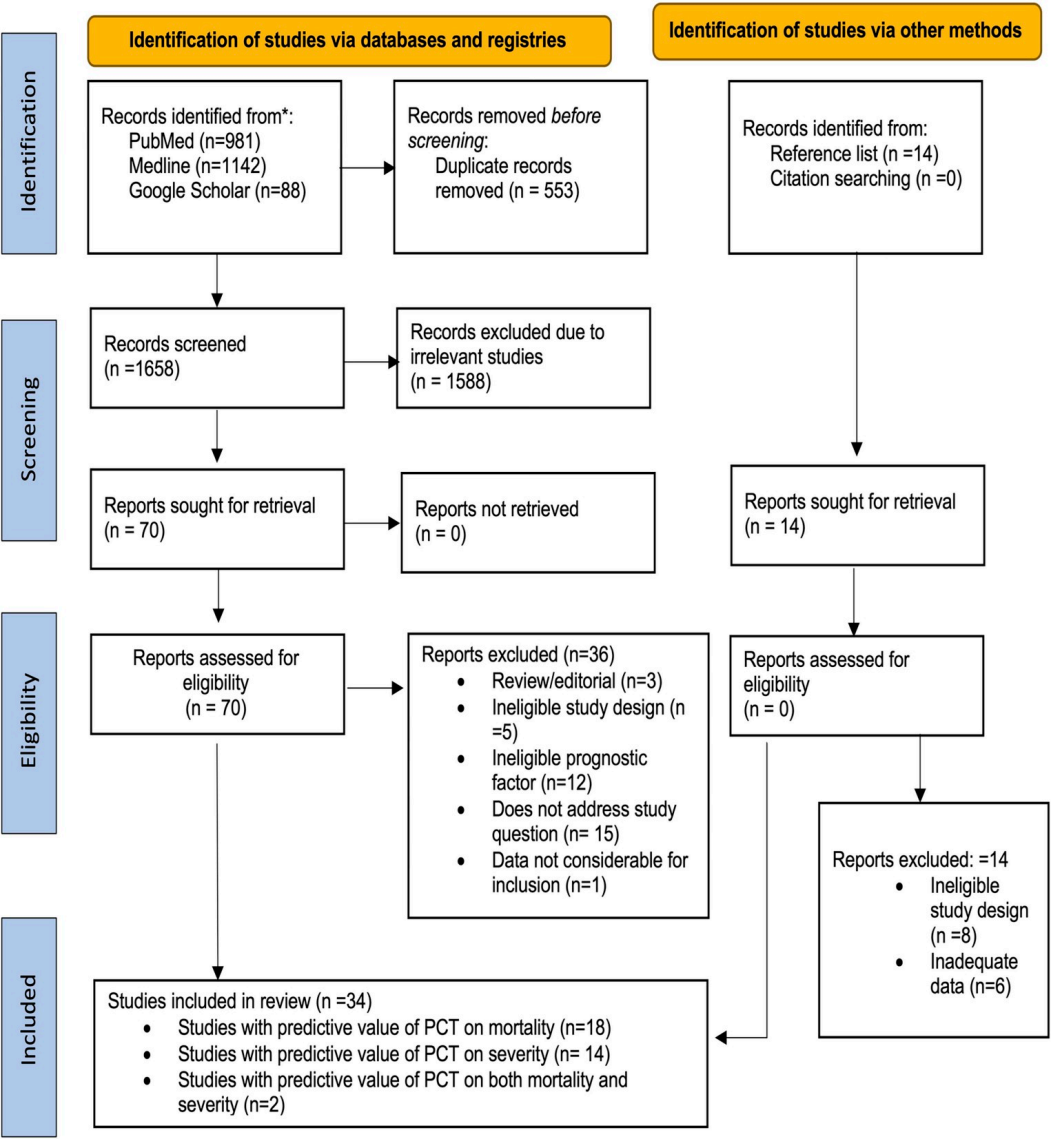
Study flow diagram (PRISMA) representing the study selection and inclusion.

In this study, 32 studies with a total sample size of 13,154 reported adequate information for computing the prognostic significance of PCT in COVID-19 patients [Fig 1].

Average age of the patients ranged from 44 years to 72 years, and the maximum average length of hospital stay was 20 days. Sixteen studies provided data for the prognosis of patients undergoing critical situations leading to mortality [21–36], and fourteen studies [4, 11, 37–48]provided data for analysis to predict only severity. In addition, two studies [49, 50] reported data for both, mortality and severity of COVID-19. Twenty-one studies were reported from China [4, 10–12, 25, 26, 28, 29, 31, 34, 36, 38, 39, 41–47, 49], two from USA [23, 41], and two from Spain [24, 38] one from Pakistan [22], one from Belgium [34], one from UK [28], one from Switzerland [27], one from Turkey [30], one from Algeria [49], and one from Italy [31]. Study characteristics are presented in Table 1. Reasons for exclusion of potential studies assessed for full text are available in the S1 Table.

### 3.2. Methodological quality assessment

**Fig 2:**
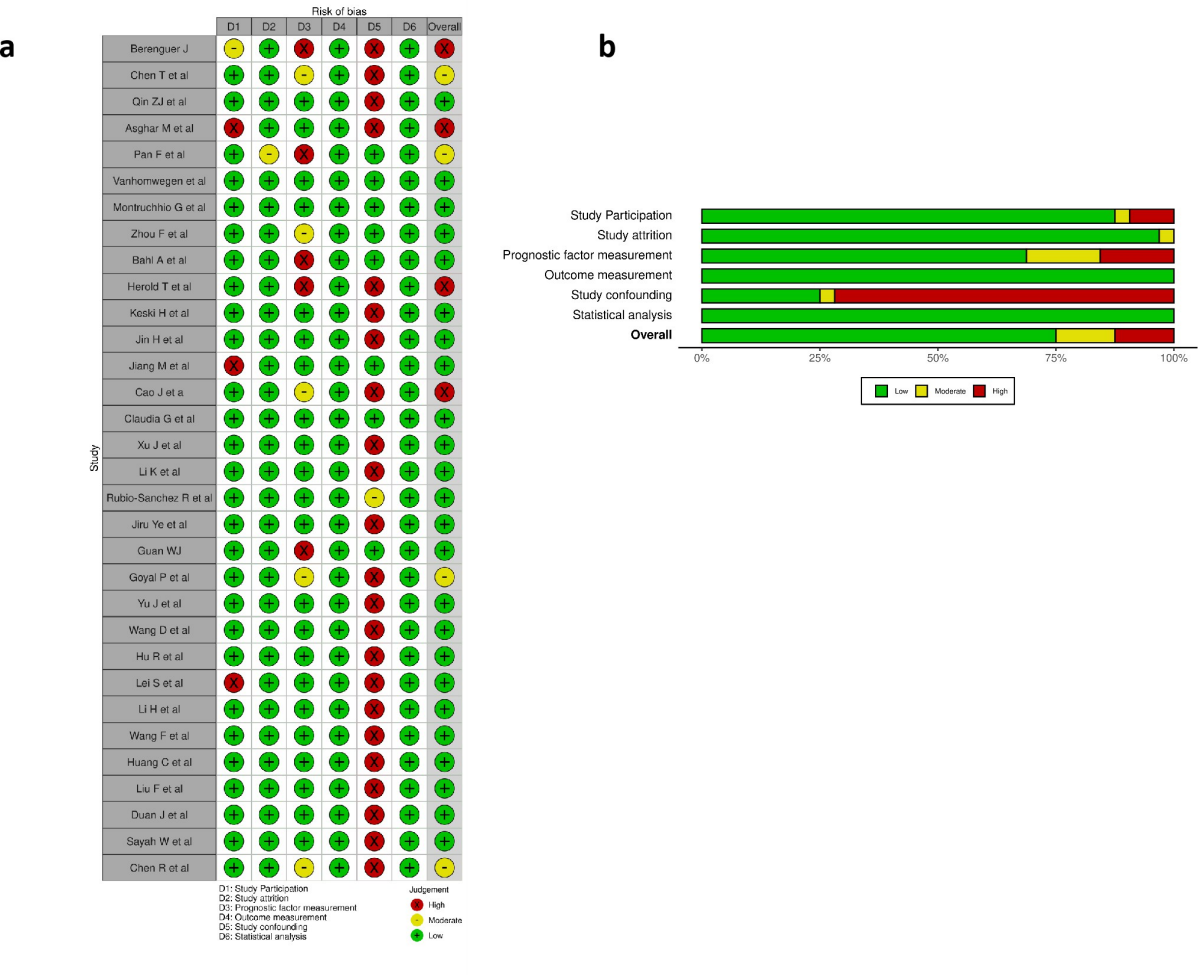
Risk of bias analysis using QUIPS tool for studies included in the meta-analysis. a. Risk of bias for individual studies; b. Summary risk of bias for each domain.

Out of the 32 studies, 24 studies had a high risk of bias in the confounding domain mainly due to failure to conduct blood culture to exclude the presence of bacterial infection. We found five studies at high risk of bias in the prognostic factor measurement domain mainly due to inadequate samples with PCT data and due to unavailability of PCT assay method. In the study participation domain, three studies were classified as having a high risk of bias; the rest were judged to have a moderate risk of bias, mainly due to insufficient information and poorly defined inclusion and exclusion criteria. We evaluated all the studies at low risk for attrition, outcome measurement, and statistical analysis domains. Overall, we judged four studies at high risk of bias, four at moderate risk of bias, and twenty-four studies at low risk of bias [Fig 2A and 2B].

### 3.3. Predictive value of PCT on mortality

**Fig 3:**
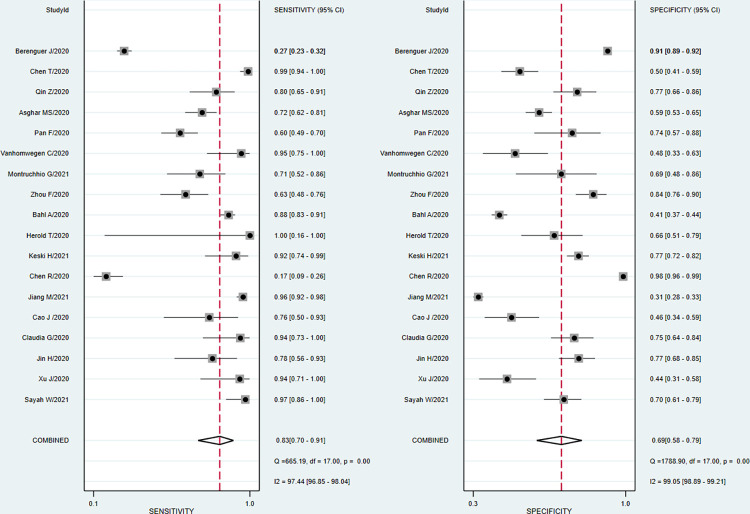
Forest plot of the pooled sensitivity and pooled specificity of PCT in overall studies for the prediction of mortality in COVID-19 patients.

A total of eighteen studies involving 9,905 patients reported sufficient data for computing pooled sensitivity and pooled specificity for mortality. Our pooled analysis observed a clinically implacable prognostic value of PCT for the prediction of mortality in COVID-19 patients. The pooled sensitivity, specificity, and diagnostic odds ratio for predicting mortality were 0.83 (95% CI: 0.70 to 0.91), 0.69 (95% CI: 0.58 to 0.79), and 11 (95% CI: 7 to 17) respectively [Fig 3].

**Fig 4:**
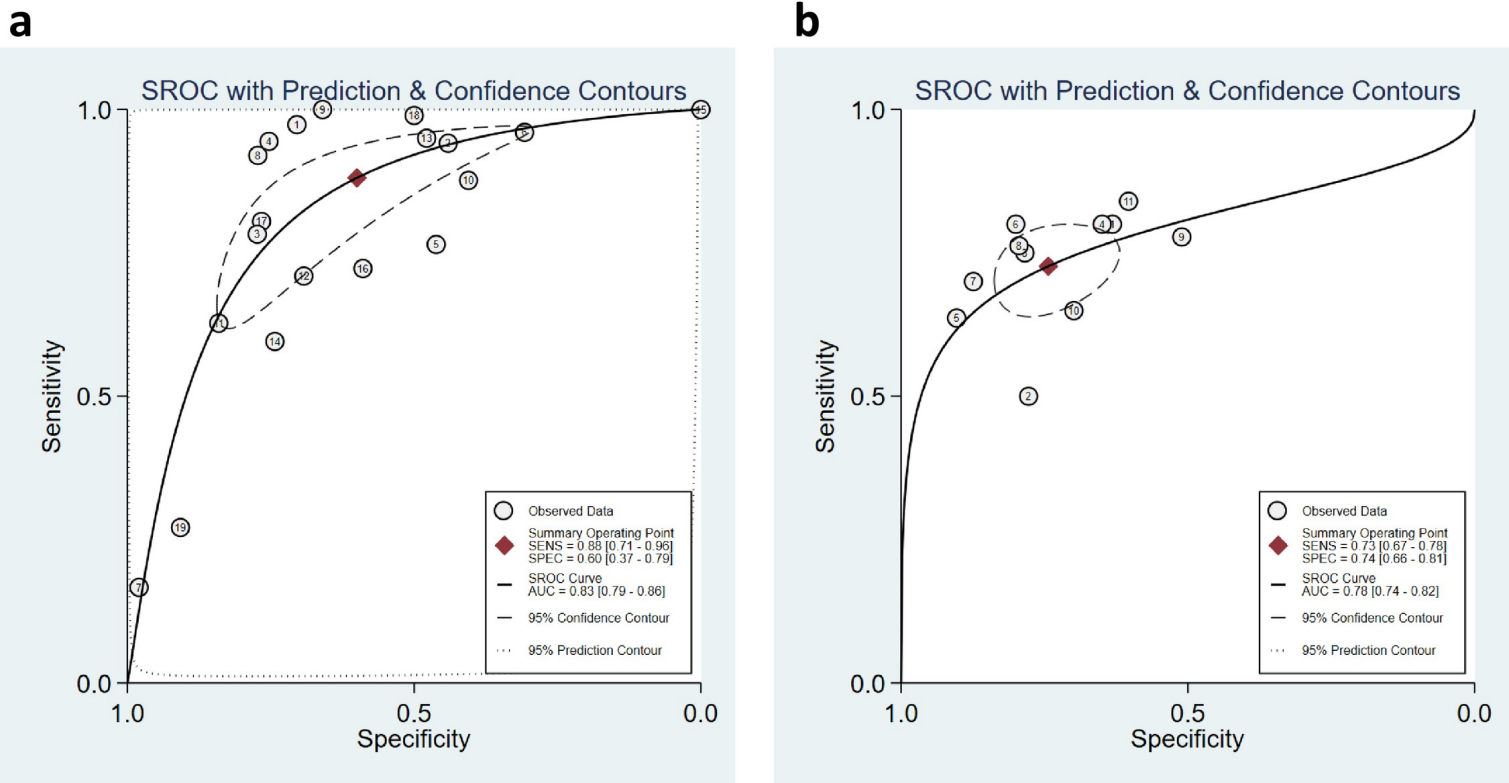
a. Summary ROC curve with prediction and confidence contours shows the discriminatory power of PCT for mortality in overall studies; b. Summary ROC curve with prediction and confidence contours shows the discriminatory power of PCT for the disease severity in sensitivity studies.

The discriminatory power of PCT for mortality was clinically acceptable, depicted by promising sAUC at 0.83 (95% CI: 0.79 to 0.86) [Fig 4A]. In the sensitivity analysis, after excluding two potentially outlier studies [24, 50], we noted that the pooled sensitivity, pooled specificity, and sAUC were 0.87 (0.79 to 0.93), 0.63 (0.54 to 0.71) and 0.81 (0.78 to 0.85) respectively [S1 Fig].

**Fig 5:**
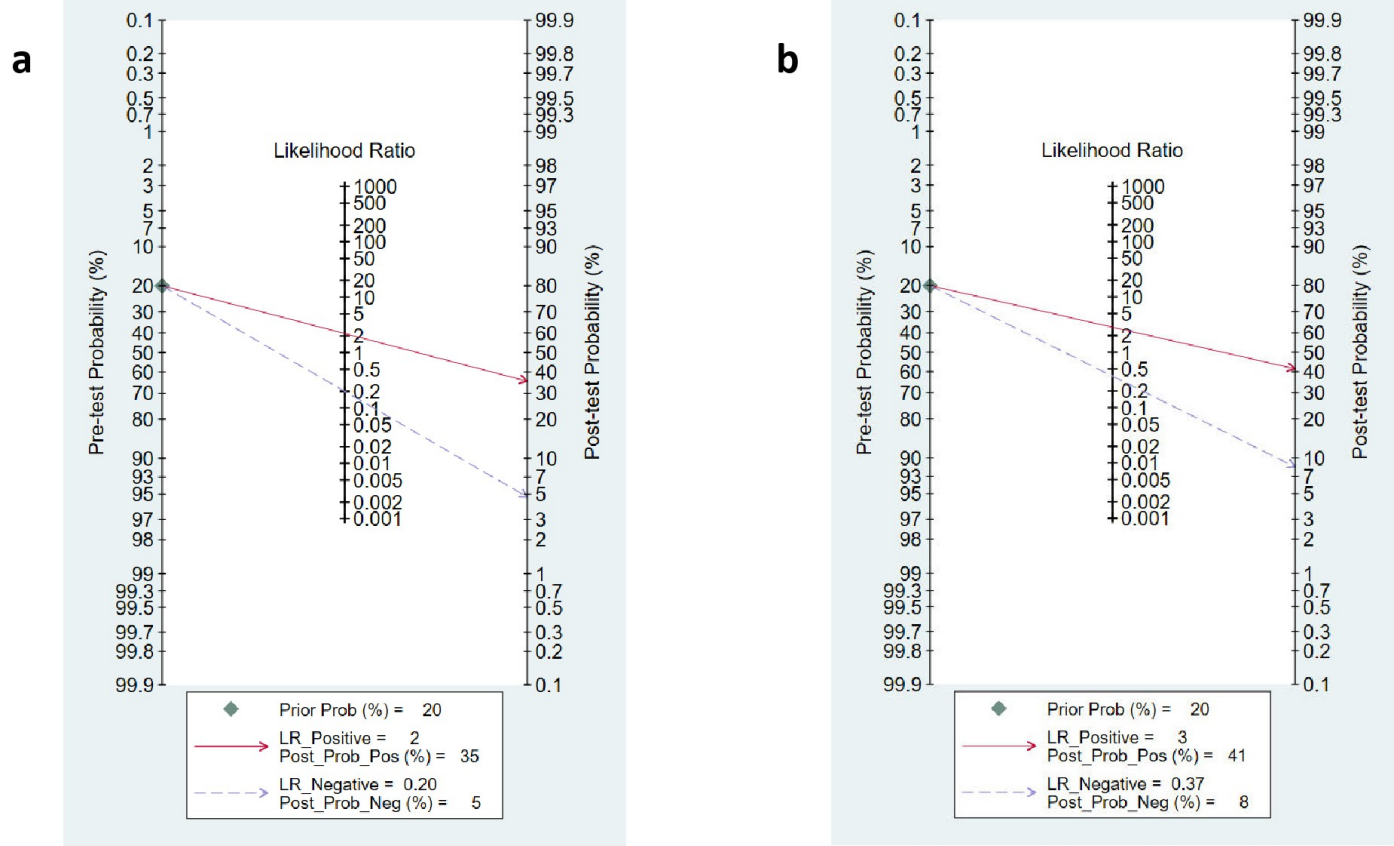
Fagan nomogram of PCT. a. Nomogram analysis showing pre-test and post-test probability of PCT for the prediction of mortality in overall studies in patients with COVID-19; b. Nomogram analysis showing pre-test and post-test probability of PCT for the prediction of severity in sensitivity studies in COVID-19 patients.

Nomogram analysis was done by setting the pre-test probability of PCT at 20%. The post-test probability for the detection of mortality was 35% when PCT was above the cut-off value. The post-test probability was 5% when the PCT was below the cut-off value [Fig 5A]. The positive and negative likelihood ratios were 2.7 (95% CI 2.1 to 3.5) and 0.25 (95% CI 0.16 to 0.41) respectively. Our findings are further strengthened by the lack of publication bias (P = 0.06) [S5 Fig]. We observed substantially high heterogeneity (I^2^ = 97.4% in sensitivity and 99.05% for specificity), and the proportion of heterogeneity likely due to threshold effect was 0.66%.

**Fig 6:**
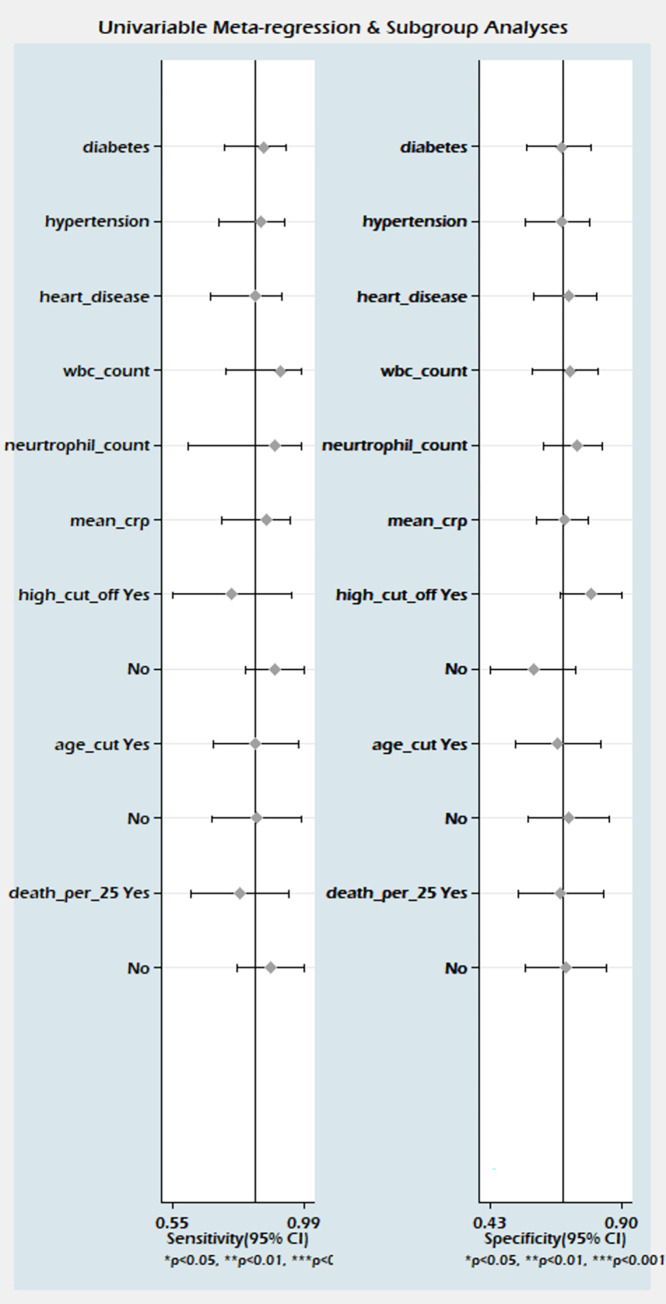
Meta-regression analysis showing the potential sources of heterogeneity on pooled effect size in studies predicting mortality.

We explored the source of heterogeneity using clinically important variables (diabetes mellitus, hypertension, cardiac disease, total WBC count, neutrophil count, CRP, and cut-off value of PCT) on the effect size. However, we did not find any of the variables significantly explaining the source of variation on pooled sensitivity and pooled specificity [Fig 6].

In our subgroup analysis, we analyzed the data in the subgroups of PCT cut-off values less than or equal to 0.10 ng/ml and PCT more than 0.10 ng/ml. The analysis again did not explain the heterogeneity significantly [Table 2].

**Table 2:** Results of subgroup analysis of PCT for predicting mortality and severity in COVID-19 patients.

Categories	Sensitivity	Specificity	sAUC	Diagnostic Odds Ratio	I^2^
**Prediction of mortality (all studies)**					
Cut-off ≤ 0.10 (n = 8)	0.88(0.79 to 0.94)	0.58(0.45 to 0.71)	0.82(0.78 to 0.85)	10(6 to 18)	Sen: 92.9% Spe: 98.3%
Cut-off > 0.10 (n = 10)	0.92(0.41 to 0.99)	0.57(0.14 to 0.92)	0.85(0.81 to 0.87)	15(4 to 55)	Sen: 99.4% Spe: 99.7%
≤25% mortality (n = 9)	0.87(0.71 to 0.95)	0.70(0.51 to 0.85)	0.87(0.83 to 0.89)	16(8 to 33)	Sen: 97.9% Spe: 99.2%
>25% morality (n = 9)	0.91(0.52 to 0.99)	0.48(0.14 to 0.83)	0.79(0.75 to 0.82)	9(4 to 22)	Sen: 99.2% Spe: 99.6%
Age ≤ 61.5 years (n = 9)	0.83(0.62 to 0.93)	0.71(0.53 to 0.85)	0.84 [0.80–0.87]	12(5 to 26)	Sen: 93.7% Spe: 96.4%
Age > 61.5 years (n = 9)	0.82(0.65 to 0.92)	0.67(0.52 to 0.79)	0.80 [0.77–0.84]	10(5 to 17)	Sen: 98.5% Spe: 99.4%
**Prediction of mortality (sensitivity analysis)**					
Cut -off ≤ 0.10 (n = 8)	0.88(0.79 to 0.94)	0.58(0.45 to 0.71)	0.82(0.78 to 0.85)	10(6 to 18)	Sen: 92.2% Spe: 98.3%
Cut-off > 0.10 (n = 8)	0.89(0.73 to 0.96)	0.68(0.60 to 0.76)	0.80(0.76 to 0.83)	17(5 to 56)	Sen: 88.3% Spe: 84.6%
≤25% mortality (n = 8)	0.92(0.86 to 0.97)	0.62(0.49 to 0.73)	0.90(0.87 to 0.92)	19(9 to 40)	Sen: 87.9% Spe: 98.8%
>25% morality (n = 8)	0.82(0.68 to 0.91)	0.64(0.51 to 0.74)	0.78(0.74 to 0.81)	8(4 to 15)	Sen: 92% Spe: 94.7%
**Prediction of severity (all studies)**					
Cut off≤0.10 (n = 9)	0.68(0.51 to 0.81)	0.81(0.72 to 0.87)	0.83(0.79 to 0.86)	9(5 to 15)	Sen: 89% Spe: 88%
Cut off >0.10 (n = 7)	0.45(0.22 to 0.71)	0.87(0.73 to 0.94)	0.78(0.74 to 0.81)	5(3 to 9)	Sen: 97.8% Spe: 98.3%
≤35% severity (n = 9)	0.61(0.41 to 0.78)	0.81(0.69 to 0.89)	0.79(0.75 to 0.82)	7(4 to 10)	Sen: 94.4% Spe: 97.2%
>35% severity (n = 7)	0.56(0.31 to 0.78)	0.86(0.73 to 0.93)	0.82(0.79 to 0.85)	7(4 to 13)	Sen: 99.4% Spe: 99.7%
**Prediction of severity (sensitivity analysis)**					
Cut-off ≤0.1 (n = 7)	0.74(0.65 to 0.81)	0.77(0.68 to 0.84)	0.81(0.77 to 0.84)	9(6 to 15)	Sen: 22.9% Spe: 82.4%
Cut-off >0.1 (n = 4)	0.70(0.63 to 0.77)	0.74(0.58 to 0.85)	0.74(0.70 to 0.78)	7(3 to 14)	Sen: 43.39% Spe: 93.7%
≤35% severity (n = 6)	0.74(0.65 to 0.81)	0.72(0.59 to 0.82)	0.79(0.75 to 0.82)	7 (4 to 12)	Sen: Spe: 88.3%
>35% severity (n = 5)	0.71(0.65 to 0.76)	0.79(0.71 to 0.85)	0.79(0.75 to 0.82)	9 (5 to 17)	Sen: 50.9% Spe: 68.1%

Abbreviations: DOR, diagnostic odds ratio; sAUC, summary area under the curve; Sen, sensitivity; Spe, specificity.

The source of variability was not explained in another subgroup analysis based on the mortality in individual studies, less than or equal to 25% and more than 25% [Table 2]. The validity of the findings is also supported by the Well-fit model by the Goodness of fit test, bivariate normality, and outlier detection test [S7 Fig]. Only one study published by Chen R et al. had a significant influence [S7 Fig].

### 3.4. Predictive value of PCT on severity

**Fig 7:**
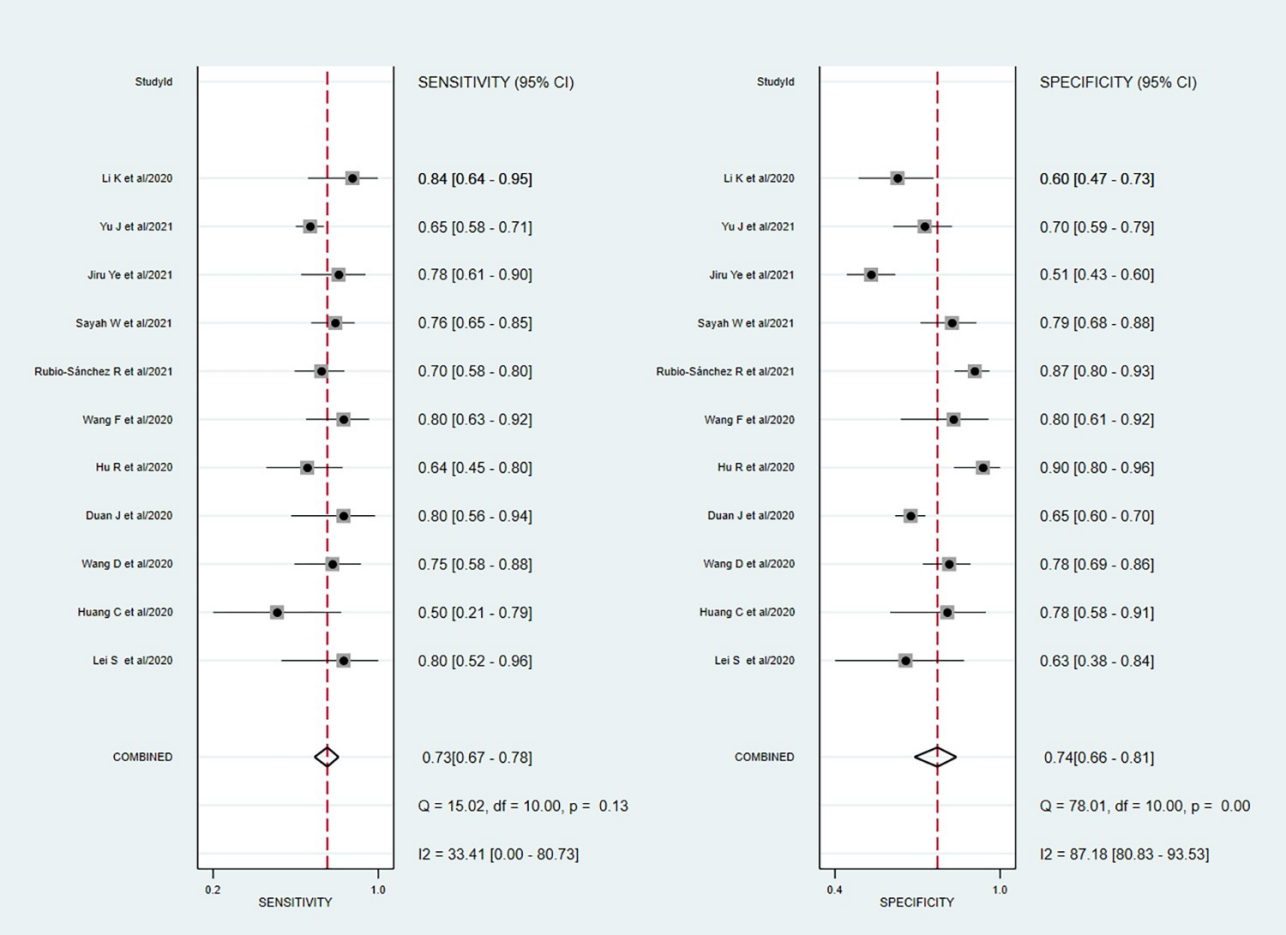
Forest plot of the pooled sensitivity and a pooled specificity of PCT in sensitivity studies for the prediction of disease severity in COVID-19 patients.

A total of sixteen studies involving 3950 patients reported adequate data for the pooled analysis. However, five studies [4, 40, 41, 45, 50] with change in the directionality of effect were removed in the sensitivity analysis, and finally, eleven studies were analyzed for determining the pooled sensitivity and specificity of PCT for predicting severity. The analysis observed good diagnostic performance of PCT for the prediction of disease severity in COVID-19 patients with pooled sensitivity 0.73 (95% CI 0.67 to 0.78); pooled specificity 0.74 (95% CI 0.66 to 0.81); and sAUC 0.78 (0.74 to 0.82) respectively [Figs 7 and 4B].

**Fig 8:**
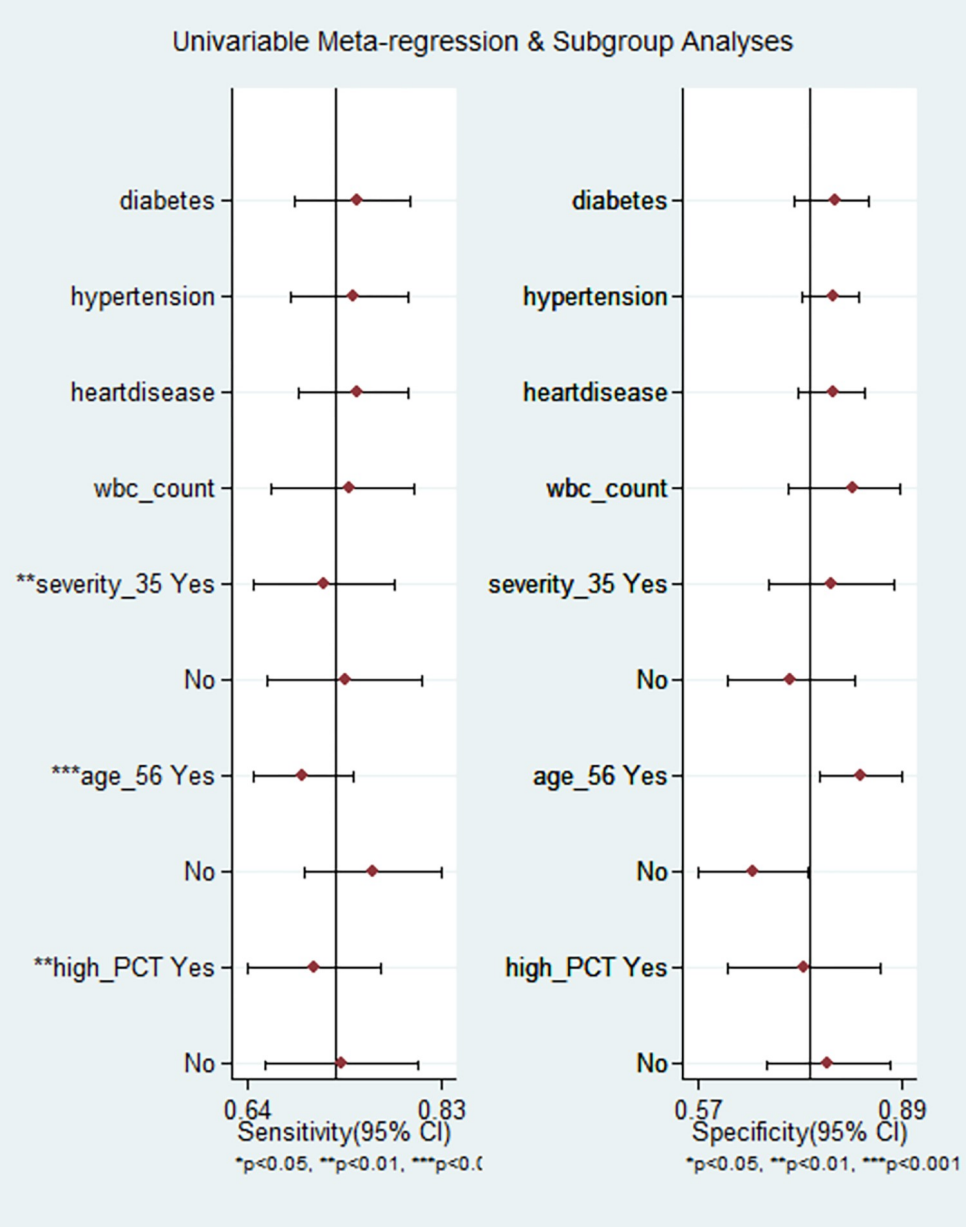
Meta-regression analysis showing the potential sources of heterogeneity on pooled effect size in studies predicting severity.

The heterogeneity was in lower range for sensitivity (I^2^ = 33.4), but considerable heterogeneity was noted for specificity (I^2^ = 87.2). The proportion of heterogeneity likely due to the threshold effect was 38%. The diagnostic odds ratio was 8.0 (95% CI: 5 to 12.0). The positive and negative likelihood ratios were 2.8 (95% CI 2.1 to 3.7) and 0.37 (95% CI 0.30 to 0.45). By setting the pre-test probability of disease severity at 20%, the nomogram analysis suggested that elevated PCT level may increase the post-test probability for the detection of severe cases to 41% [Fig 5B]. Similarly, if the pre-test probability for a favorable outcome was set at 20%, the post-test probability for negative outcome decreased to 8% with a normal level of PCT [Fig 5B]. Our findings are supported by the lack of publication bias (P = 0.89) as seen in the Deeks funnel plot asymmetry test [S6 Fig]. The proportion of diabetes, hypertension, heart disease, total WBC count, and mean age did not explain the source of heterogeneity on pooled effect size in the meta-regression analysis. However, a significant influence of PCT cut-off values, Severity proportion > 35% and mean age >56 years in the individual studies were the source of variation on pooled effect size in the meta-regression analysis [Fig 8].

The validity of the findings is supported by the Well-fit model by the Goodness of fit test, bivariate normality, and outlier detection test [S8 Fig].

### 3.5. Subgroup analysis

We conducted subgroup analyses based on the cut-off value in the studies predicting mortality and severity. In eleven out of total 32 studies, the authors used cut-off based on ROC curves whereas 21 studies used pre-defined cut-off values. While predicting mortality, the cut-off value in nine studies was more than 0.1 ng/ml and this was termed the “high cut-off value” subgroup. Nine studies used a cut-off value of ≤ 0.1 ng/ml and were included in the “low cut-off value” subgroup. The sensitivity and specificity in “low cut-off value” sub-group were 0.92 (95% CI 0.83–0.97) and 0.72 (95% CI 0.57–0.83) respectively. Similarly, in the “high cut-off value subgroup, the sensitivity, and the specificity for predicting mortality were 0.89 (CI 0.79–0.94) and 0.67 (CI 0.58–0.75), respectively. Subgroup analysis of the studies involving disease severity was performed using the same cut-off values. Out of the eleven studies predicting disease severity, seven studies had a PCT cut-off value of ≤ 0.1 ng/ml, whereas four studies had a cut-off value >0.1 ng/ml. There were six studies with severity defined as per Chinese Clinical Guidance [51], four studies with severity defined by ICU admission, and one study with severity defined by WHO interim guidance for COVID-19 [52]. We performed subgroup analysis based on the severity defined by Chinese Clinical Guidance and on the severity defined by ICU admission criteria. Considerable heterogeneity was noted in the studies which defined severity based on Chinese clinical guidelines [sensitivity (I^2^ = 52.88%) and for specificity (I^2^ = 86.5%)]. Lesser heterogeneity was observed in the studies using severity criteria based on ICU admission [for sensitivity (I^2^ = 12.91%), and for specificity (I^2^ = 62.7%)]. Median age was ≤ 61.5 years in nine of the 18 studies predicting mortality whereas in another nine studies it was > 61.5 years. Subgroup analysis was done based on median age group of the participants for the prediction of mortality in the studies included in the meta-analysis. No significant difference in predictive value was observed between ≤ 61.5 years and > 61.5 years age group. Detailed results of subgroup analysis and sensitivity analysis for predicting mortality and severity are presented in Table 2.

### 3.6. Grade analysis

GRADE profiling showed the low quality of evidence for sensitivity and specificity of PCT in predicting mortality. However, in the case of severity prediction, we observed a moderate level of certainty in the evidence for sensitivity and very low certainty of the evidence for specificity using PCT as a biomarker [S2 and S3 Tables].

## 4. Discussion

The present meta-analysis highlighted the predictive accuracy of PCT for mortality and disease severity in COVID-19 patients using a robust methodology and recommended guidelines involving 13,154 participants from 32 studies for obtaining clear evidence.

Our study demonstrated that PCT predicts mortality (AUC = 0.83, SEN = 0.83, and SPE 0.69) and disease severity (AUC = 0.78, SEN = 0.73 and SPE = 0.74) in COVID-19.

PCT appears to be a reliable biomarker for predicting outcomes and adapting appropriate therapeutic care in patients with COVID-19.PCT is widely used to predict the severity of lower respiratory tract disease, sepsis, and mortality due to bacterial infection [54, 55]. However, growing research suggests that severe respiratory infection is associated with a rise in PCT level even in the absence of bacterial infection [56]. Studies have recently reported that mildly elevated procalcitonin levels help in the prognostication of COVID-19. A high PCT level at the time of admission might indicate an inflammatory response, leading subsequently to immune hyperactivation and cytokine storm. However, bacterial superinfection is very difficult to assess in patients of severe COVID-19.

The prognostic significance of PCT in COVID-19 patients is addressed in the literature via systematic reviews and meta-analyses; however, their findings are constrained by several limitations. Ahmed S et al. [12] in their systematic review, evaluated the role of PCT for predicting the severity of COVID-19 in 52 research articles, but they could not perform a meta-analysis due to the lack of uniformity of the statistical models in the studies. Lippi et al. [10]in their meta-analysis included only four studies, whereas the meta-analysis by Shen et al. [14] included ten studies which led to insufficient power to provide credible evidence. A meta-analysis by Zare et al. [13] that included 16 studies stated that among the six biomarkers studied, only PCT was found to be a promising biomarker for predicting severity and mortality. However, the authors did not assess the methodological quality of studies using the QUIPS tool, GRADE criteria for the certainty of the evidence, publication bias, and subgroup analysis, all of which could have limited the study findings. This meta-analysis also includes an extreme outlier study by Mikami T et al. [57] which had a high sample size (2126 individuals) and reported no patients with true negative and false-negative readings, which is very rare in biomarker predictive studies. The foregoing flaw might have again contributed to bias in the pooled analysis. Also, the study did not examine the optimal cut-off value of PCT in detail for prognostic significance. None of the meta-analyses have examined the cut-off value of PCT for predicting mortality or severity. The current meta-analysis overcomes these limitations by employing a larger number of studies (32 studies involving 13,154 participants) and adhering to the established guidelines for reporting meta-analysis results of prognostic studies. Table 3 shows comparison of the current meta-analysis with previous meta-analyses and the add-on value to the current literature.

**Table 3:** Comparison of the current meta-analysis with previous meta-analyses.

Criteria		Lippi G; 2020	Shen Y, 2021	ZareME; 2020	Pesent meta-analysis
Number of studies		4	10	16	35
Number of participants		1,417	7,716	12,209	15,974
Recommended guidelines for prognostic meta-analysis reporting	Pooled sensitivity	×	×	√	√
	Pooled sensitivity	×	×	√	√
	Summary Area under the curve	×	×	√	√
	Diagnostic odds ratio	×	×	√	√
	Methodological quality (QUIPS)	×	×	×	√
	GRADE criteria	×	×	×	√
	Publication Bias	×	√	×	√
Analysis used		Pooled odds ratio.	Pooled odds ratio	Pooled sensitivity, Pooled specificity, Summary Area under the curve, Diagnostic odds ratio.	Pooled sensitivity, Pooled specificity, Summary Area under the curve, Diagnostic odds ratio.

Our meta-analysis revealed the pooled prognostic significance of procalcitonin in predicting mortality and severity of COVID-19 using pooled sensitivity, pooled specificity, the summary area under the curve, publication bias, GRADE evidence, QUIPS tool for the assessment of the risk of bias, and model validity test. The findings of our meta-analysis indicate that PCT could be used as a promising prognostic marker for the prediction of mortality in COVID-19 patients with 83% sensitivity and 69% specificity and for prediction of severity with 73% sensitivity and 74% specificity. As per the pooled DOR [11 (95% CI: 7 to 17) and 8 (95% CI: 5 to 12) respectively] in our study, PCT has higher accuracy for the prognosis of mortality in comparison to critical condition. Our findings are supported by the meta-analysis by Zare et al. [13] which gave a pooled DOR of 13.21 (3.95–44.19) and 6.78 (3.68–33.15) respectively for mortality and disease severity due to COVID-19. There was considerable variation in the outcomes across the studies in our meta-analysis. Upon examination of heterogeneity in the meta-regression analysis, we could not find any clinical or biochemical variable (mean age, prevalence of diabetes and hypertension, total WBC count, neutrophil count, CRP, or inclusion of serious patients in the study population) that could account for the heterogeneity in the mortality prediction. However, in case of severity prediction, our meta-regression analysis showed considerably less heterogeneity in the subgroup of studies that had less than 35% severity rate (I^2^ = 31.9% for sensitivity) and among studies that considered cut–off value ≤ 0.10 (I^2^ = 22.9% for sensitivity). We found no significant difference in the heterogeneity between the studies which had younger population with a mean age of ≤ 61.5 years and those with elder population with a mean age > 61.5 years. The summary area under the curve for the studies with mean age ≤61.5 for discriminating mortality was slightly higher than in the <61.5 years subgroup. However, it was statistically not significant (P = 0.11).

The cut-off values for mortality prediction ranged from 0.05 ng/mL to 0.5 ng/mL, and for severity prediction ranged from 0.04 ng/mL to 0.795 ng/ml. In our subgroup analysis, we found no significant difference in discriminating power between studies with a cut-off value of ≤0.10 and studies with a cut-off value of > 0.10 for the prediction of mortality in COVID-19 patients [Table 2]. On the other hand, for prediction of severity, we observed increased predictive accuracy in the subgroup of studies that used a cut-off value of 0.10 (sAUC, 0.83), compared to the studies that used a cut-off value greater than 0.10 (sAUC, 0.78). Following sub-group analysis, our study suggests that a cut-off value of 0.1 ng/ml may be used for predicting mortality and severity at the time of admission in patients with COVID-19. Patients with PCT values more than 0.1 ng/ml may be offered intensive treatment early in the course of the disease to save their lives, and their treatment may be optimized accordingly. Furthermore, a PCT value of more than 0.5 ng/mL is considered as an indication of systemic bacterial or fungal infection [55].The viral infection leads to interferon-gamma production, which in turn leads to inhibition of procalcitonin production from various tissues [58]. Therefore, lower cut-off values were observed in the included studies. The dynamicity of PCT in blood is reflected by PCT induction within two to four hours after the onset of bacterial infection, reaching peak levels at 24–48 hours and a decline with a disapperance rate of 50% of approximately 24 to 36 hours [59]. However, due to insufficient information in the included studies, we could not extract the data about time interval between clinical presentation and blood sampling. Future studies should report the data on time interval to discuss the influence of timing of PCT assay since clinical presentation for the prediction of mortality and severity in COVID-19 patients.

Several limitations should be noted when considering the findings of the present meta-analysis. Most of the studies were retrospective in design and were monocentric giving rise to subjectivity of information. Some studies reported higher cut-off value limiting the optimal cut-off values, for its clinical use. However, 75% of the studies reported PCT cut-off values below 0.16 ng/ml suggesting that the optimal cut-off value may lie between 0.05 and 0.16. Statistical and clinical heterogeneities were substantially high. Differences in the clinical settings, ethnicity, time of admission since clinical presentation, methods of the measurement of PCT, bias in study conduct due to unblinded PCT analysis, presence of unstudied confounding variables, use of different cut-off values of PCT, use of different measures of severity and presence of interstitial pneumonia might have introduced bias in the study results. The models used in individual studies were not validated for precise evidence. Because many studies did not use blood cultures to rule out bacterial infection, the findings of this meta-analysis might have confounded the study results. Finally, the last search of articles in our study was conducted up to May 31, 2021, and the manuscripts published from June 2021 till now could not be included in this meta-analysis.

## 5. Conclusion

This study demonstrates that PCT overexpression can strongly predict poor outcomes in COVID-19 patients; evaluating PCT at the time of admission may help clinicians identify potentially severe cases early. Nevertheless, further well-designed, and high methodological quality multi-centric studies from different populations are needed to obtain precise predictive accuracy of PCT for mortality and disease severity in COVID-19 patients. Future studies should report the predictive accuracy of PCT with a predefined cut-off value at 0.1 ng/ml to obtain homogenous findings.

## Supporting information

S1 Checklist (DOCX)

S1 Table Reason for exclusion of potential studies (assessed full text articles). (DOCX)

S2 Table GRADE analysis for the certainty of evidence for the sensitivity and specificity of PCT in predicting mortality. (DOCX)

S3 Table GRADE analysis for the certainty of evidence for the sensitivity and specificity of PCT in predicting severity. (DOCX)

S1 Fig Forest plot of the pooled sensitivity and the pooled specificity of PCT for the prediction of mortality in sensitivity studies in COVID-19 patients. (TIF)

S2 Fig Forest plot of the pooled sensitivity and the pooled specificity of PCT in overall studies for the prediction of disease severity in COVID-19. (TIF)

S3 Fig Summary ROC curve with prediction and confidence contours shows the discriminatory power of PCT for mortality in sensitivity studies. (TIF)

S4 Fig Summary ROC curve with prediction and confidence contours shows the discriminatory power of PCT for the disease severity in all studies. (TIF)

S5 Fig Deeks funnel plot asymmetry test for publication bias for the prediction of mortality. (TIF)

S6 Fig Deeks funnel plot asymmetry test for publication bias for the prediction of severity. (TIF)

S7 Fig Well-fit model by the Goodness of fit test, bivariate normality, and outlier detection test for the studies predicting mortality. (TIF)

S8 Fig Well-fit model by the Goodness of fit test, bivariate normality, and outlier detection test for the studies predicting severity. (TIF)
