# Supplementary material for: Procalcitonin as a predictive marker in COVID-19: A systematic review and meta-analysis
Source: PLoS One. 2022 Sep 9;17(9):e0272840. doi: 10.1371/journal.pone.0272840 (PMC9462680; doi:10.1371/journal.pone.0272840)
Supplement: S1 Table — (DOCX) [file pone.0272840.s002.docx]

**S1 Table. Reason for exclusion of potential studies (assessed full text articles)**

| **Study no.** | **Author, Year** | **Reason for Exclusion** | **Key finding** |
| --- | --- | --- | --- |
| 1. | Su W, 2020 | Sensitivity & specificity was not reported | Neutrophilia, thrombocytopenia, hypersensitive troponin I greater than 0.04 pg/mL and procalcitonin more than 0.1 ng/mL may he the potential risk factors to identify patients with poor prognosis at an early stage. |
| 2. | Muhammad R , 2020 | Sensitivity & specificity was not reported | Patients of advanced age that present with elevated biomarkers of inflammation, coagulation, and end-organ damage were at higher risk of mortality.  . |
| 3. | Dolci A, 2021 | The cut-off was higher than the maximum considered one. | PCT testing did not provide a significant added value when compared with more common markers of infection. The possible application of PCT in COVID-19 lies in its ability to exclude a bacterial co-infection when a cutoff for ruling out is applied |
| 4. | Ding Ronrong, 2020 | Sensitivity & specificity was not reported | Serum CRP and FDP levels are positively related to the severity of COVID-19. The finding indicates that CRP and FDP levels may potentially be used as early predictors for severe illness and help physicians triage numerous patients in a short time |
| 5. | Krause M, 2020 | Sensitivity & specificity was not reported | Association between an initial plasma procalcitonin level >0.1 ng/ml and the duration of mechanical ventilation. This helps to identify patients at risk for prolonged mechanical ventilation upon admission. |
| 6. | Cecconi M, 2020 | Sensitivity and specificity was not reported | Age, respiratory rate, and lab parameters including CRP and PCT were predictors of clinical deterioration. A prognostic index tool was developed to assess prognosis. |
| 7. | Nicholson CJ, 2020 | Sensitivity & specificity was not reported | The chronic use of a statin was associated with protection against death due to COVID-19 |
| 8. | McRae MP, 2020 | Sensitivity & specificity was not reported. | A clinical decision tool was developed to predict mortality in COVID-19. |
| 9. | Yu C, 2020 | Multimodal specificity & sensitivity was reported | Older age, male sex, history of diabetes, lymphopenia, and increased PCT on admission had significant associations with COVID-19 mortality |
| 10. | Chen X, 2020 | No death/ alive or severe/ non-severe records were found | PCT levels to differentiated COVID‐19 patients from control patients. |
| 11. | Hong Y, 2020 | No death/ alive or severe/ non-severe records were found | Elevated PCT was a strong and independent predictor of ProLOS (hospitalization for more than 14 days) |
| 12. | Zhang B, 2020 | No death/ alive or severe/ non-severe records for PCT were found | To quantify the severity of 2019 novel coronavirus disease (COVID-19) on chest CT and to determine its relationship with laboratory parameters |
| 13. | Wang CZ, 2020 | No death/ alive or severe / non-severe records for PCT were found | Neutrophilia is a laboratory independent risk factor for the progression of COVID-19 pneumonia. |
| 14. | Yang A, 2020 | No death/ alive or severe/ non-severe records for PCT were found. | SARS-CoV-2 infection may be associated with cellular immunodeficiency, myocardial and hepatic injury, nutrient consumption and hypoxemia. Compared with patients with mild symptoms, those with severe or critical symptoms exhibited differences in neutrophil count and CD4 expression. |
| 15. | Hou H, 2020 | No death/ alive or severe / non-severe records for PCT were found | The IL-2R/lymphocyte was a prominent biomarker for early identification of severe COVID-19 and predicting the clinical progression of the disease. |
| 16. | Mikami T, 2020 | Data not considerable for inclusion; No true negative and false negative subjects. | Older age, male sex, hypotension, tachypnea, hypoxia, impaired renal function, elevated D-dimer, and elevated troponin were associated with increased in-hospital mortality in COVID-19 patients. |
