# Supplementary material for: Procalcitonin as a predictive marker in COVID-19: A systematic review and meta-analysis
Source: PLoS One. 2022 Sep 9;17(9):e0272840. doi: 10.1371/journal.pone.0272840 (PMC9462680; doi:10.1371/journal.pone.0272840)
Supplement: S2 Table — (DOCX) [file pone.0272840.s003.docx]

**S2 Table. GRADE analysis for the certainty of evidence for the sensitivity and specificity of PCT in predicting mortality.**

**Question**: Should [PCT] be used to diagnose [mortality] in [COVID-19]?

| \| Sensitivity \| 0.83 (95% CI: 0.70 to 0.91) \| \| --- \| --- \| \| Specificity \| 0.69 (95% CI: 0.58 to 0.79) \| |  | \| Prevalences \| 20% \| 25% \| 30% \| \| --- \| --- \| --- \| --- \| |  |
| --- | --- | --- | --- | --- | --- | --- | --- | --- | --- | --- | --- |

| Outcome | № of studies (№ of patients) | Study design | Factors that may decrease certainty of evidence | | | | | Effect per 1,000 patients tested | | | Test accuracy CoE |
| --- | --- | --- | --- | --- | --- | --- | --- | --- | --- | --- | --- |
|  |  |  | Risk of bias | Indirectness | Inconsistency | Imprecision | Publication bias | pre-test probability of 20% | pre-test probability of 25% | pre-test probability of 30% |  |
| **True positives** (patients with [mortality]) | 18 studies 6761 patients | cross-sectional (cohort type accuracy study) | not serious | not serious | very serious | not serious | none | 166 (140 to 182) | 208 (175 to 228) | 249 (210 to 273) | ⨁⨁◯◯ Low |
| **False negatives** (patients incorrectly classified as not having [mortality]) |  |  |  |  |  |  |  | 34 (18 to 60) | 42 (22 to 75) | 51 (27 to 90) |  |
| **True negatives** (patients without [mortality]) | 18 studies 6761 patients | cross-sectional (cohort type accuracy study) | not serious | not serious | very serious | not serious | none | 552 (464 to 632) | 518 (435 to 593) | 483 (406 to 553) | ⨁⨁◯◯ Low |
| **False positives** (patients incorrectly classified as having [mortality]) |  |  |  |  |  |  |  | 248 (168 to 336) | 232 (157 to 315) | 217 (147 to 294) |  |
