# Supplementary material for: Procalcitonin as a predictive marker in COVID-19: A systematic review and meta-analysis
Source: PLoS One. 2022 Sep 9;17(9):e0272840. doi: 10.1371/journal.pone.0272840 (PMC9462680; doi:10.1371/journal.pone.0272840)
Supplement: S3 Table — (DOCX) [file pone.0272840.s004.docx]

**S3 Table. GRADE analysis for the certainty of evidence for the sensitivity and specificity of PCT in predicting severity.**

**Question**: Should [PCT] be used to diagnose [target Severity] in [COVID-19]?

| \| Sensitivity \| 0.73 (95% CI: 0.67 to 0.78) \| \| --- \| --- \| \| Specificity \| 0.74 (95% CI: 0.66 to 0.81) \| |  | \| Prevalences \| 25% \| 30% \| 35% \| \| --- \| --- \| --- \| --- \| |  |
| --- | --- | --- | --- | --- | --- | --- | --- | --- | --- | --- | --- |

| Outcome | № of studies (№ of patients) | Study design | Factors that may decrease certainty of evidence | | | | | Effect per 1,000 patients tested | | | Test accuracy CoE |
| --- | --- | --- | --- | --- | --- | --- | --- | --- | --- | --- | --- |
|  |  |  | Risk of bias | Indirectness | Inconsistency | Imprecision | Publication bias | pre-test probability of 25% | pre-test probability of 30% | pre-test probability of 35% |  |
| **True positives** (patients with [target Severity]) | 11 studies 3418 patients | cross-sectional (cohort type accuracy study) | not serious | serious | not serious | not serious | none | 183 (168 to 195) | 219 (201 to 234) | 256 (235 to 273) | ⨁⨁⨁◯ Moderate |
| **False negatives** (patients incorrectly classified as not having [target Severity]) |  |  |  |  |  |  |  | 67 (55 to 82) | 81 (66 to 99) | 94 (77 to 115) |  |
| **True negatives** (patients without [target Severity]) | 11 studies 3418 patients | cross-sectional (cohort type accuracy study) | not serious | serious | very serious | not serious | none | 555 (495 to 608) | 518 (462 to 567) | 481 (429 to 527) | ⨁◯◯◯ Very low |
| **False positives** (patients incorrectly classified as having [target Severity]) |  |  |  |  |  |  |  | 195 (142 to 255) | 182 (133 to 238) | 169 (123 to 221) |  |
