# Supplementary figures and images for: Procalcitonin as a predictive marker in COVID-19: A systematic review and meta-analysis
Source: PLoS One. 2022 Sep 9;17(9):e0272840. doi: 10.1371/journal.pone.0272840 (PMC9462680; doi:10.1371/journal.pone.0272840)

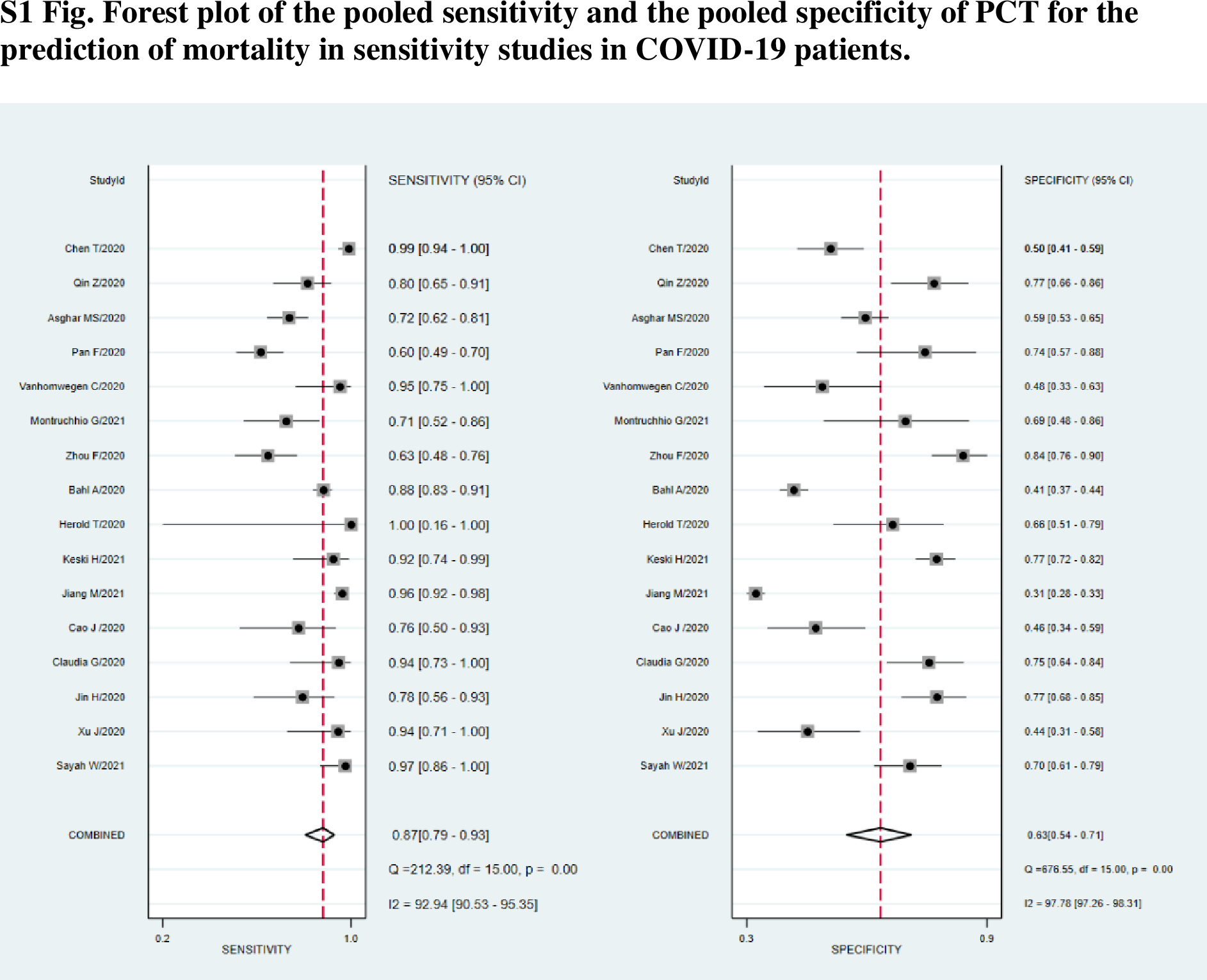

Supplement: S1 Fig — (TIF) [file pone.0272840.s005.tif]

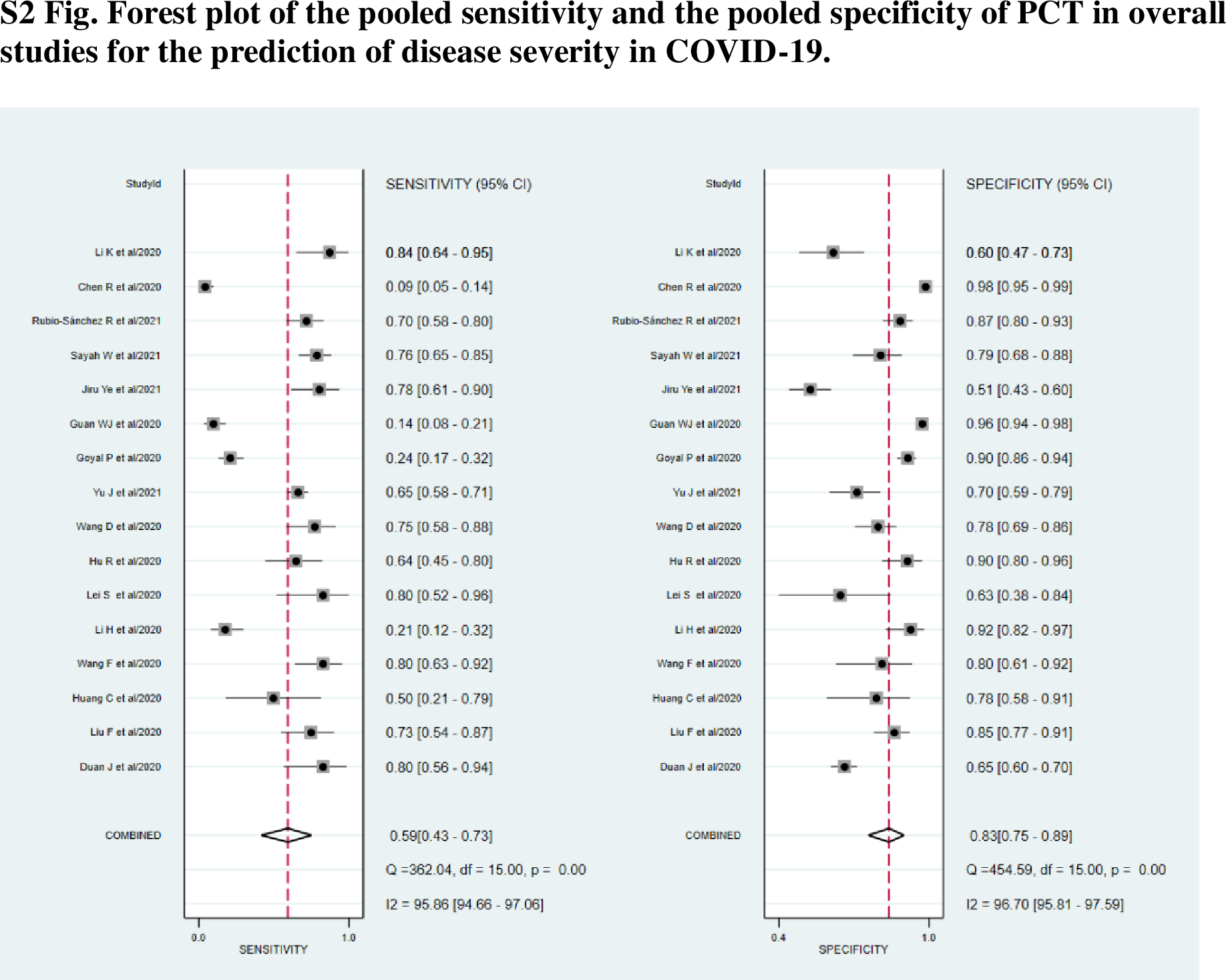

Supplement: S2 Fig — (TIF) [file pone.0272840.s006.tif]

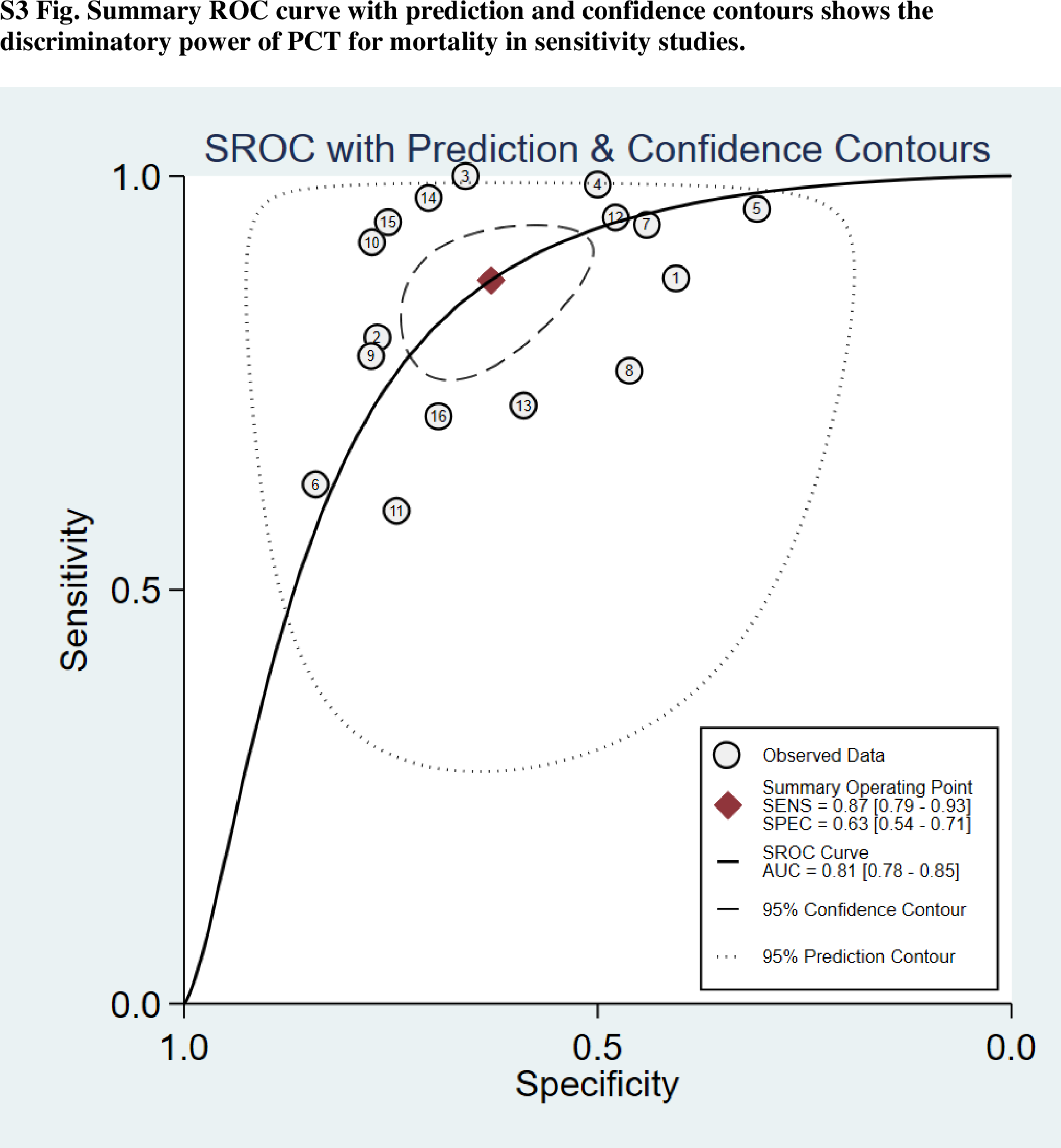

Supplement: S3 Fig — (TIF) [file pone.0272840.s007.tif]

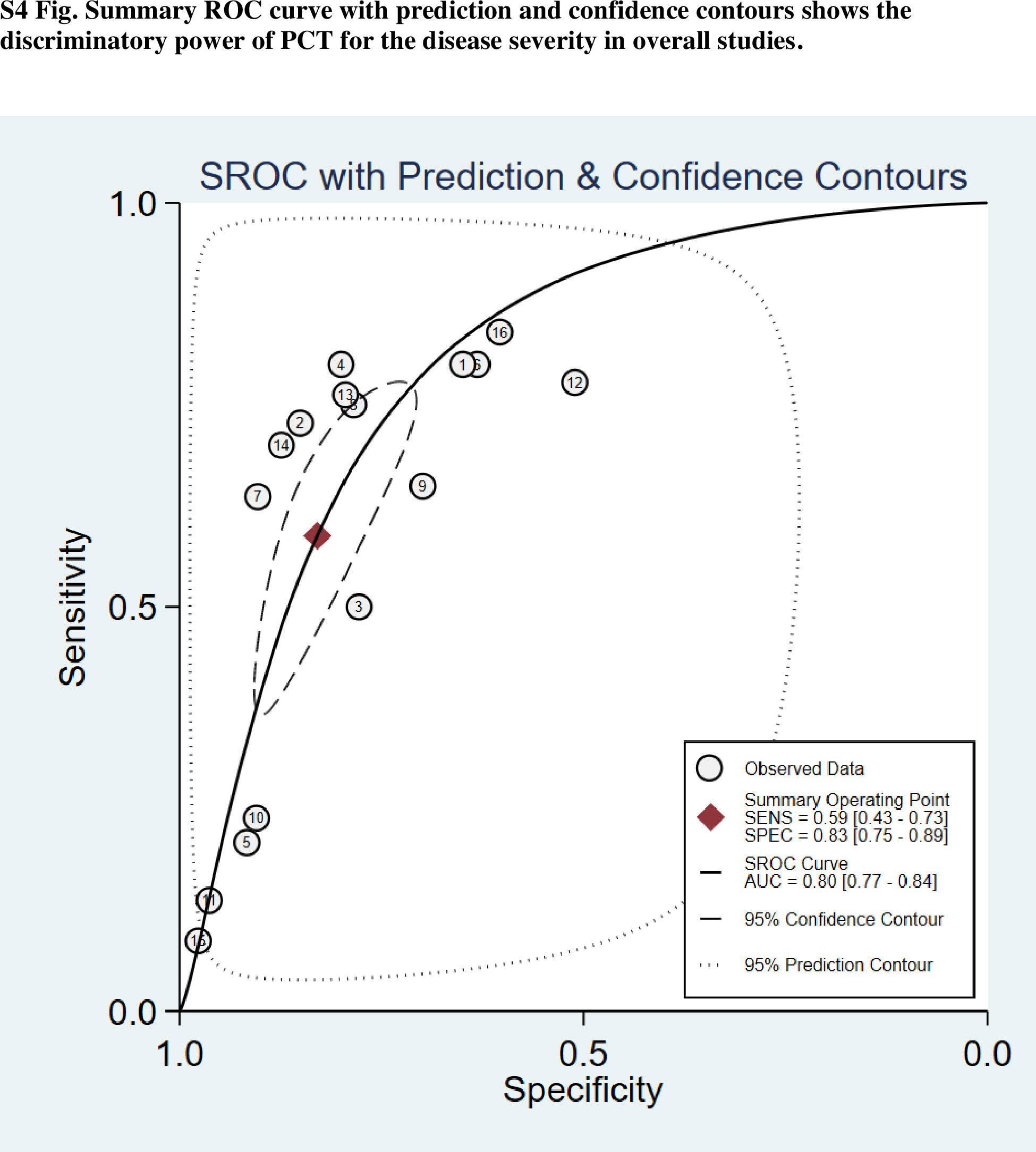

Supplement: S4 Fig — (TIF) [file pone.0272840.s008.tif]

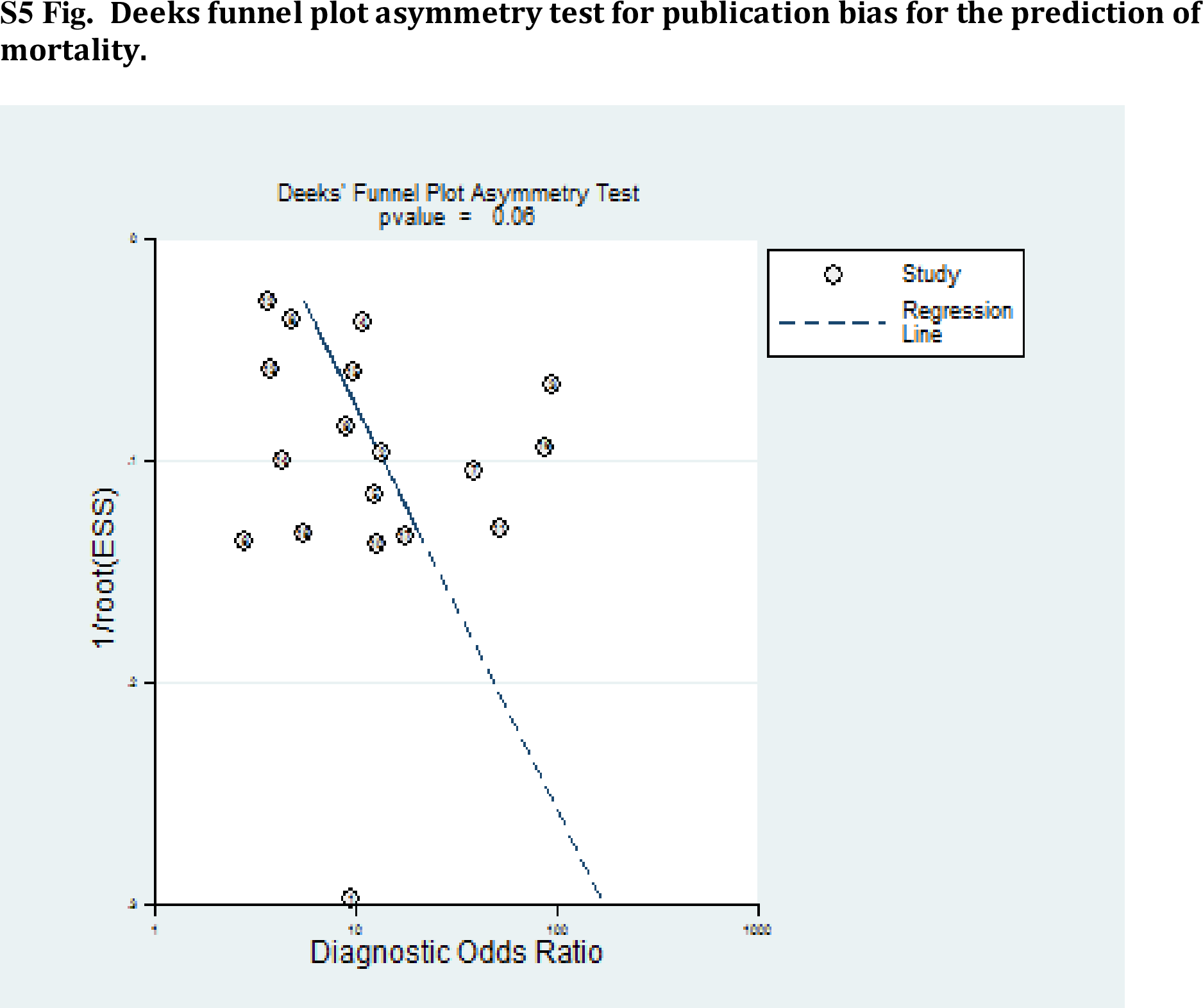

Supplement: S5 Fig — (TIF) [file pone.0272840.s009.tif]

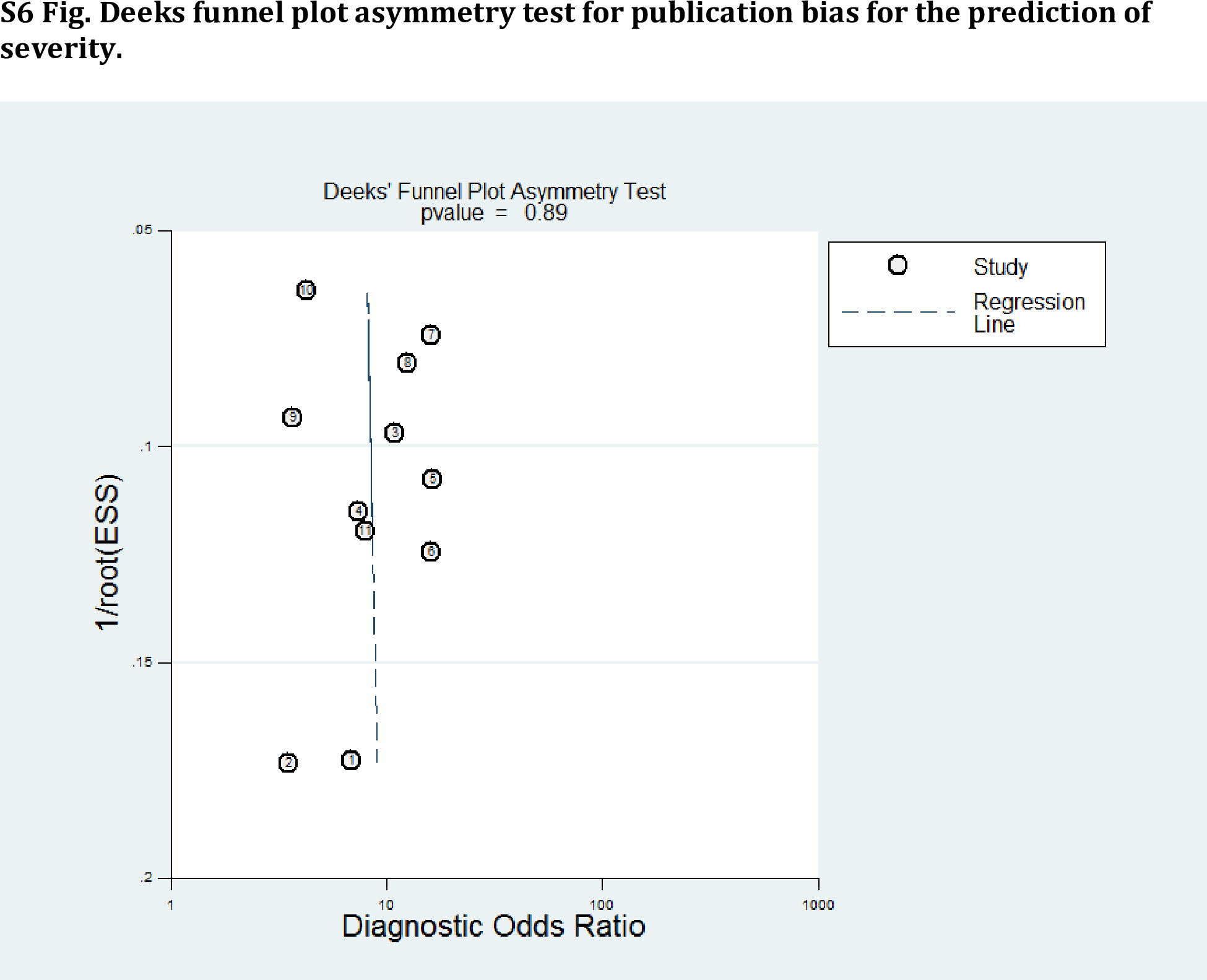

Supplement: S6 Fig — (TIF) [file pone.0272840.s010.tif]

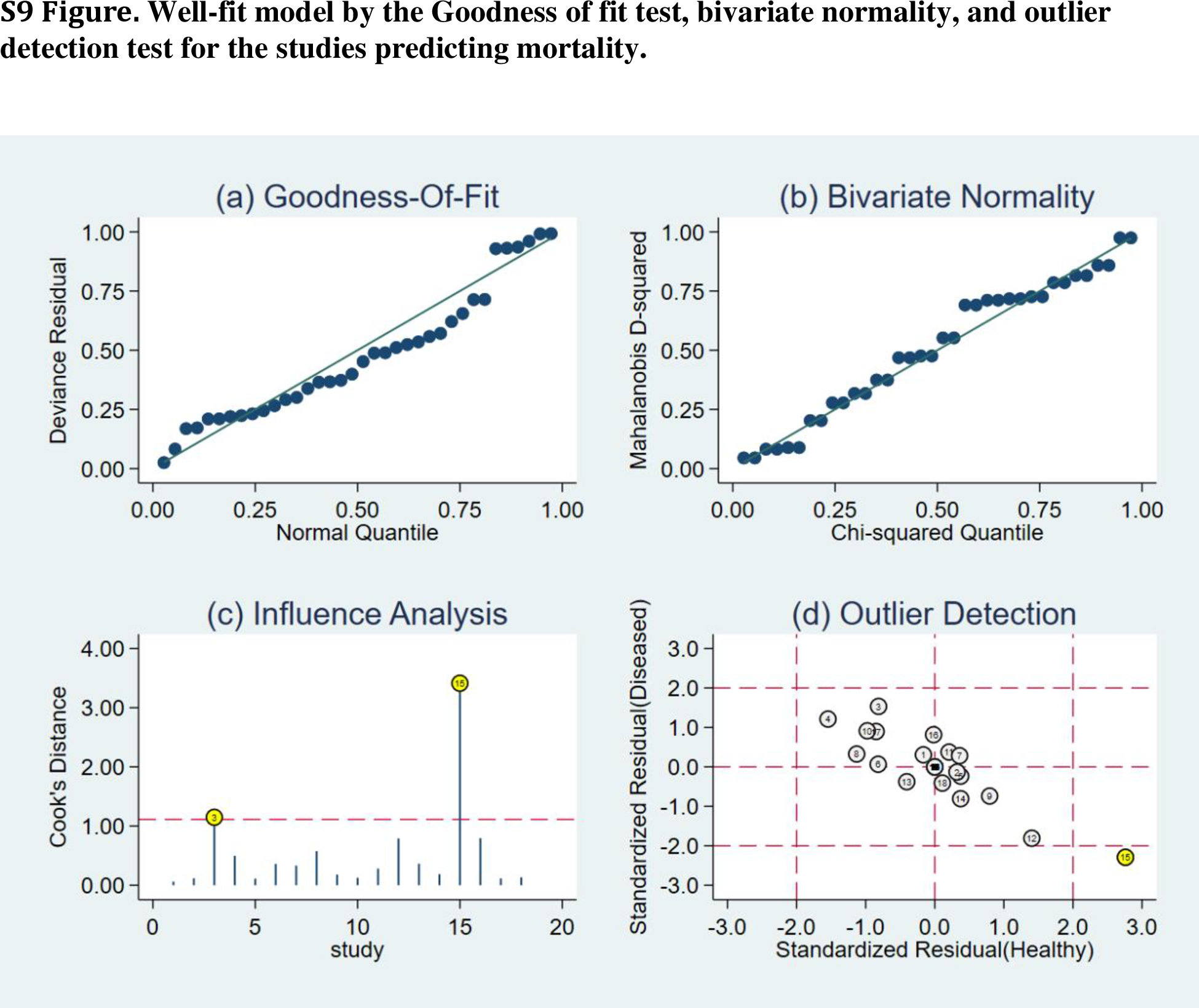

Supplement: S7 Fig — (TIF) [file pone.0272840.s011.tif]

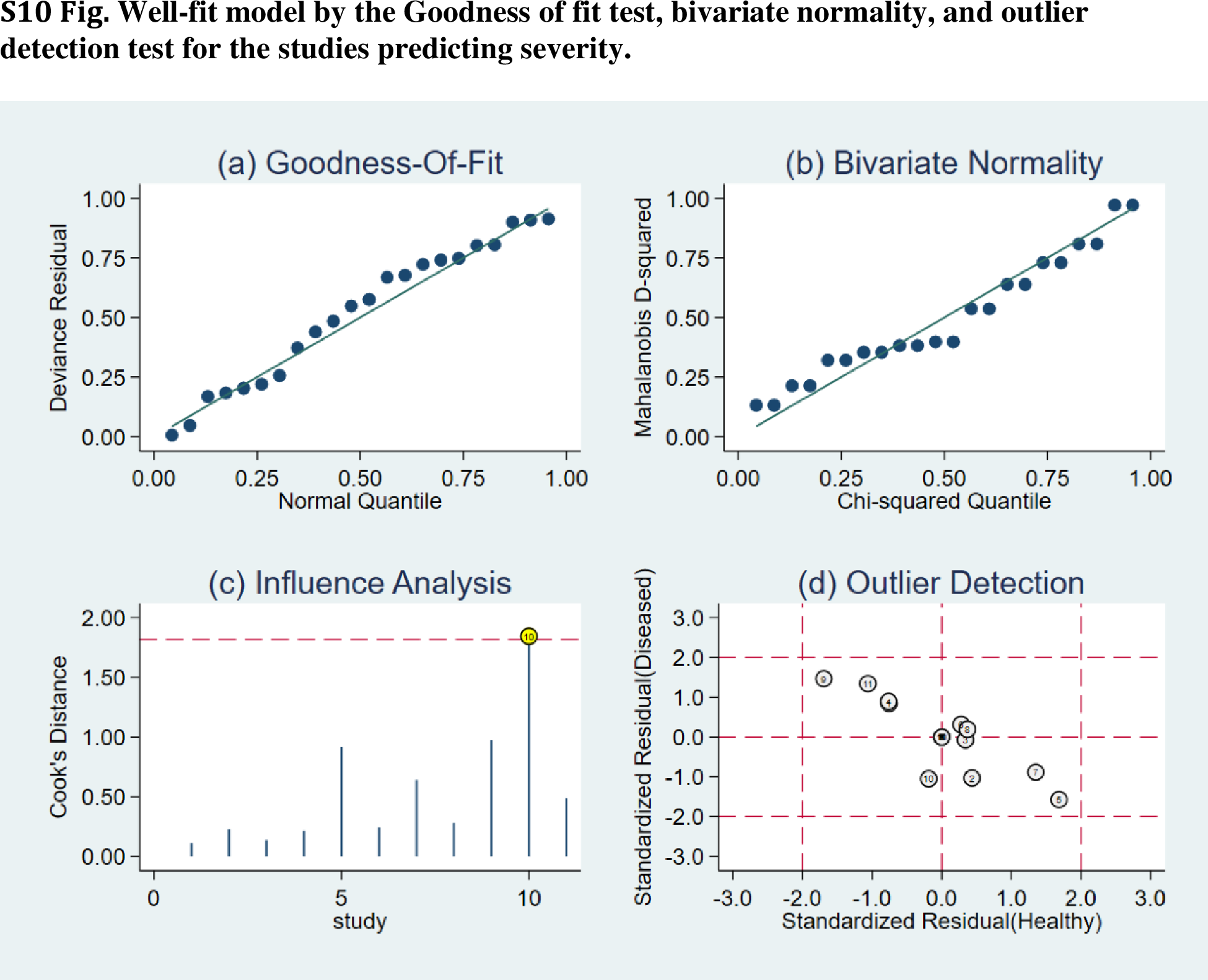

Supplement: S8 Fig — (TIF) [file pone.0272840.s012.tif]
